# Development of Long-Term Stable MXene-Based Gas Sensing Material

**DOI:** 10.3390/molecules30224440

**Published:** 2025-11-17

**Authors:** Jiabin Yang, Qingfu Dai, Haodong Wu, Li Yang, Shenghui Guo, Qiuni Zhao, Ming Hou, Sridhar Komarneni, Yi Xia

**Affiliations:** 1 School of Metallurgical and Energy Engineering, Kunming University of Science and Technology, Kunming 650093, China; yanglikmust@163.com (L.Y.); shguo78@hotmail.com (S.G.); qiunizhao90s@163.com (Q.Z.); houmingkmust@163.com (M.H.); 2 Faculty of Materials Science and Engineering, Kunming University of Science and Technology, Kunming 650093, China; yangjiabin2025@163.com (J.Y.); daiqingfu0077@163.com (Q.D.); 19185635889@163.com (H.W.); 3 Research Center for Analysis and Measurement, Kunming University of Science and Technology, Kunming 650093, China; 4 Department of Ecosystem Science and Management and Materials Research Institute, 204 Energy and the Environment Laboratory, The Pennsylvania State University, University Park, PA 16802, USA

**Keywords:** MXene, long-term stable, gas sensor

## Abstract

In recent years, rapid industrial development has led to the emission of diverse gaseous pollutants into the atmosphere. To detect and monitor these pollutants, gas sensors have become a critical technology. Researchers have developed numerous gas-sensitive materials, among which MXenes—a novel class of two-dimensional materials—have garnered significant attention. Owing to their excellent electron transport properties, abundant surface functional groups, and large specific surface area, MXenes find wide applications in catalysis, sensing, electromagnetic shielding, water treatment, and beyond. However, despite these outstanding properties, MXenes’ susceptibility to environmental degradation has hindered their broader development and application as long-term stable gas-sensitive materials. While recent studies have investigated degradation mechanisms and explored various stability enhancement strategies, comprehensive reviews specifically focusing on stability improvements for gas-sensing applications remain scarce. This review first examines the current research on MXene oxidation processes in different environments. Subsequently, it systematically summarizes existing strategies to enhance MXene’s long-term stability and its implementation in gas sensing, including optimization of preparation methods, surface protection and modification, composite construction, and other approaches. Finally, the review concludes by summarizing current progress and outlining future perspectives.

## 1. Introduction

With the continuous advancement of industrialization, an array of pollutants—particularly exhaust gases such as NO_x_, SO_2_, CO, H_2_S, and volatile organic compounds (VOCs) [1,2,3,4,5,6]—are released into the environment. These contaminants not only inflict substantial damage on natural ecosystems but also pose significant risks to human health [7]. Additionally, colorless and odorless gases frequently employed in the fossil fuel industry, including H_2_ and CH_4_, exhibit extreme flammability [8]. Notably, methane has an explosion limit ranging from 5% to 15% [9,10], while hydrogen’s explosion range spans 5% to 75% [11]. Leakage of such flammable gases can precipitate catastrophic incidents, underscoring the critical necessity of their detection and monitoring. The advent of gas sensors has revolutionized the surveillance of polluting gases, with chemiresistive gas sensors garnering extensive adoption owing to their streamlined architecture and cost-effectiveness [12,13].

**Figure 1:**
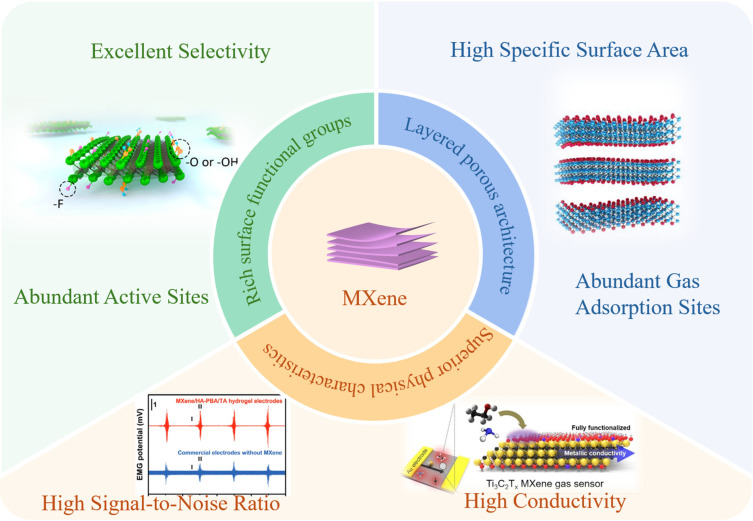
A Comprehensive Summary of the Advantages [20,35,36,37] of MXene as a Gas-Sensitive Material. Reproduced with permission [20]. Copyright 2018, American Chemical Society. Reproduced with permission [35]. Copyright 2019, American Chemical Society. Reproduced with permission [36]. Copyright 2020, Spring Nature. Reproduced with permission [37]. Copyright 2024, Wiley-VCH.

Metal oxide semiconductors (MOSs) serve as the most commonly used sensing materials in chemiresistive gas sensors, with their gas-sensing mechanism rooted in the conductivity changes in the sensitive materials within devices when exposed to reducing or oxidizing gases [13]. However, MOS-based gas sensors still suffer from inherent limitations, such as poor selectivity and sluggish response–recovery kinetics, thereby driving growing research attention toward the development of novel materials and innovative gas-sensing architectures [14]. In recent years, two-dimensional (2D) layered materials have emerged as a frontier in gas-sensing research, with promising representatives including graphene, transition metal dichalcogenides (TMDs), black phosphorus, hexagonal boron nitride (h-BN), graphitic carbon nitride (g-C_3_N_4_), MXenes, two-dimensional metal–organic frameworks (2D MOFs), and 2D covalent organic frameworks (2D COFs) [15,16,17,18,19]. Among these, MXenes outperform traditional metal oxides in key properties: their high specific surface area, superior electronic characteristics, layered porous architecture, and extremely high signal-to-noise ratio [20] effectively address the long-standing challenges of poor selectivity and slow response in MOS-based sensors. The advantages of MXene in gas sensing are shown in Figure 1. Structurally, MXenes follow the general formula M_n+1_X_n_T*_x_* [21], where M denotes transition metals, X represents carbon and/or nitrogen atoms, and T (e.g., OH, F, O) are terminal chemical groups adsorbed onto the M-layer surfaces during the etching process. These materials are synthesized by selectively etching the A-element layers from MAX phases, yielding layered structures with alternating (n + 1) “M” layers and n “X” layers [22]. The exceptional properties of MXenes have enabled their versatile applications across diverse fields, including energy storage [23], water treatment [24], flexible electronics [25,26], catalysis [27], electromagnetic shielding [28], and sensing. Specifically, their metal-like electrical conductivity, tunable bandgap, surface functional groups, and layered structure provide efficient pathways for gas diffusion while exposing abundant active sites for surface redox reactions [15,29]. Notably, the practical implementation of MXenes in gas-sensing applications is currently hindered by their intrinsic susceptibility to oxidation, leading to stability issues and limited operational lifespan under harsh environmental conditions. Therefore, enhancing the long-term stability of MXenes has become a critical priority for advancing their real-world deployability.

In recent years, numerous review studies have been conducted on the preparation methods, antioxidant properties, and gas-sensing applications of MXenes. For instance, the Kruger group systematically reviewed the research progress of molten salt-etching in the preparation and modification of MXenes, while also summarizing their application scenarios [30]. Scholars such as Cao and Soomro have comprehensively reviewed the oxidation stability and antioxidant strategies of MXenes [31,32]. Additionally, Xia, Li, and others have summarized recent research findings focusing on the synthesis processes, structural characteristics, and gas-sensing properties of MXene materials [33,34]. Although existing studies have covered several important aspects of MXenes, a review specifically addressing long-term stable MXene gas-sensing materials remains absent. This review focuses on the recent research progress in long-term stable MXene gas sensors. First, the oxidation and hydrolysis mechanisms of MXenes are introduced, and the current studies on the processes and influencing factors of MXene oxidation and hydrolysis are systematically compiled, aiming to enhance the understanding of these degradation processes and develop corresponding strategies to improve MXene stability based on the underlying mechanisms. Subsequently, the strategies reported in recent years for enhancing the long-term stability of MXenes are systematically presented, mainly including the optimization of preparation methods, surface protection, composite construction, annealing treatment, elemental doping, improvement of storage conditions, etc. Finally, the future prospects of long-term stable MXene-based gas sensing materials are discussed.

## 2. Factors Affecting MXene Degradation

In the field of gas sensors, the stability of sensitive materials is a critical factor determining the performance and service life of sensors, directly affecting the accuracy and reliability of detection. MXene exhibits enormous application potential in gas sensing due to its unique electrical, mechanical, and surface properties. However, its inherent stability defects severely restrict the expansion of practical applications. MXene is extremely sensitive to environmental factors, and conditions such as water, oxygen, high temperature, and light can trigger its decomposition process. MXene decomposes rapidly when exposed to high-temperature and high-humidity environments, and even undergoes oxidation to a certain extent when stored at room temperature for a long time. Since MXene, as a sensitive material, needs to continuously contact the external environment, these degradation factors are difficult to avoid. Once degradation occurs, the excellent sensing properties of MXene, such as high sensitivity and fast response, will significantly attenuate or even completely disappear. Therefore, deeply exploring the degradation mechanism of MXene and analyzing the influence laws of key environmental factors such as water, oxygen, and high temperature on its stability has become a core topic for breaking through the technical bottleneck of MXene-based gas sensors. At present, researchers have carried out a large number of studies on these degradation triggers.

### 2.1. Oxidation Mechanism

Oxygen constitutes 21% of the atmosphere, and the main challenge in practically applying MXene to gas sensing is its oxidation resistance. Therefore, it is essential to understand the oxidation mechanism of MXene. Currently, researchers have studied the oxidation behavior of MXene in various environments.

Liu et al. [38] utilized atomic-scale in situ aberration-corrected environmental transmission electron microscopy (AC-ETEM) and reactive molecular dynamics (MD) simulations to investigate the initial oxidation of HF-etched Ti_3_C_2_T*_x_* MXene at room temperature. During the initial oxidation stage (0–65 min) of Ti_3_C_2_T*_x_* MXene, discontinuous in situ AC-ETEM observations were performed. As oxidation progressed, Ti_3_C_2_T*_x_* was gradually oxidized to α-TiO_2_, followed by phase transformations from α-TiO_2_ to β-TiO_2_ and subsequently to γ-TiO_2_. The final oxidation products were amorphous carbon and titanium dioxide, as described by Equation (1) [39]:

Ti_3_C_2_ + O_2_ → 3TiO_2_ + C (1)

**Figure 2:**
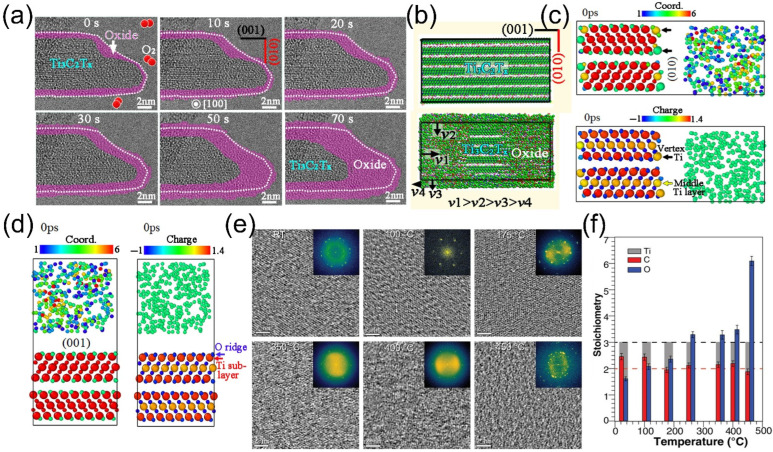
(**a**) In situ AC-HRTEM experiments demonstrating the early-stage (within 70 s) oxidation process of Ti_3_C_2_T*_x_* under continuous electron beam irradiation, with a constant flow of O_2_ gas at room temperature. (**b**) Schematic illustration depicting the characteristics of the early-stage oxidation process of the Ti_3_C_2_T*_x_* sample based on in situ observations. (**c**,**d**) Coordination structures of the (001) and (010) crystal planes before oxidation, and charge changes of atoms in the surface regions before oxidation, respectively; where the smaller spheres represent O and C, and the larger spheres represent Ti. Reproduced with permission [38]. Copyright 2024, Wiley-VCH. (**e**) Plan-view HRTEM images acquired from 1 to 3 stacked Ti_3_C_2_T*_x_* single flakes after exposure to 2 mbar O_2_ for 0.5 h, at room temperature, 100 °C, 175 °C, 350 °C, 400 °C, and 450 °C. (**f**) C/Ti/O stoichiometries obtained via EELS quantitative analysis from room temperature to 450 °C. Reproduced with permission [40]. Copyright 2020, Wiley-VCH.

Continuous AC-ETEM observations during the initial 70 s oxidation stage revealed that amorphous oxides formed incrementally from the exterior to the interior, leading to a gradual increase in the thickness of the (001) and (010) facets of the nanosheets (Figure 2a). Notably, the (010) facet exhibited a higher density of oxide formation, indicating a significantly faster oxidation rate (Figure 2b). Molecular dynamics (MD) simulations at 900 K provided further insights into this disparity. Figure 2c,d illustrate the coordination states and charge densities at the Ti_3_C_2_O_2_ (010)/O_2_ and Ti_3_C_2_O_2_ (001)/O_2_ interfaces before oxidation. Partial Ti atoms on the (010) surface (those denoted by black arrows) displayed lower coordination numbers, whereas subsurface Ti atoms on the (001) surface achieved coordinative saturation. The unsaturated bonds surrounding these low-coordination Ti atoms enabled strong interactions with O_2_, rendering them more susceptible to oxidation. Additionally, the interlayer channels within MXene expedited the diffusion of O_2_ to the (010) facet, facilitating the formation of zigzag-shaped oxides. In contrast, the (001) surface, characterized by its negatively charged outer O^−^ layer and neutral Ti subsurface, impeded oxidation due to restricted electron transfer and minimal atomic loss.

In addition to room-temperature oxidation, certain gas sensors operate at elevated temperatures. Consequently, a thorough comprehension of MXene’s high-temperature oxidation behavior is of paramount importance for its practical applications in extreme working environments. Persson et al. [40] employed high-resolution transmission electron microscopy (HRTEM), electron energy loss spectroscopy (EELS), and electron diffraction (ED) to in situ monitor the oxygen saturation process of MXene under 2 mbar O_2_, spanning from room temperature (RT) to 450 °C. HRTEM images (Figure 2e) depicted a progressive decrease in diffraction spots and an increase in diffuse transmission spots from RT to 400 °C, indicative of continuous oxidation and the formation of disordered oxidation products (validated by fast Fourier transform (FFT) insets). At 450 °C, although the two-dimensional morphology of the MXene sheets was retained, the crystalline structure completely disintegrated. EELS analysis of the C-K, Ti-L_2_,_3_, and O-K edges (Figure 2f) revealed an elevated oxygen content and charge transfer from Ti/C to O, attributed to the formation of Ti-O and C-O bonds and the reduction of Ti-C bonds. ED results demonstrated that the MXene structure remained intact below 400 °C (with no phase transformation or reaction products detected), with surface disorder arising solely from oxygen saturation. However, at 450 °C, oxygen oversaturation triggered the structural collapse, leading to the amorphization of Ti_3_C_2_T*_x_* and the formation of TiO_2_ nanoparticles, as described by Equation (2):

Ti_3_C_2_O_2_ + O_2_ → 3TiO_2_ + 2CO_2_ (2)

In the process of high-temperature oxidation, O_2_ progressively saturates the MXene surface, leading to the formation of new chemical bonds. These interactions culminate in the amorphization of the material and the subsequent development of titanium dioxide TiO_2_ nanoparticles. As MXenes become amorphous, they lose their intrinsic properties, resulting in a decline in gas-sensing performance.

### 2.2. Hydrolysis Mechanism

During their operation, gas sensors inevitably come into contact with water. Water molecules adsorb onto the surface of MXene, making it more susceptible to oxidation, which is highly detrimental to gas sensors utilizing MXene as a sensitive material. Extensive studies on the oxidation behavior of MXene in water have been conducted by researchers [41,42,43].

**Figure 3:**
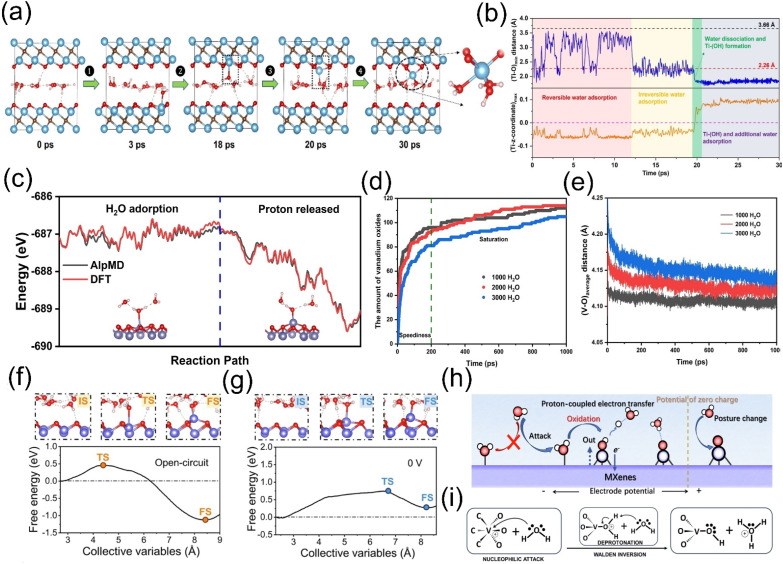
(**a**) Representative snapshots of a single water layer in Ti_3_C_2_O_2_ during 30 ps FPMD at 300 K, showing four stages of water attacking the surface. (**b**) Key distances in the simulation: top (black) dashed line is Ti-O van der Waals radii sum, bottom (red) is covalent radii sum. Reproduced with permission [44]. Copyright 2022, American Chemical Society. (**c**) Oxidation transition state energy comparison between DFT and AlpMD. (**d**,**e**) Vanadium oxide count and average V-O distance vs. time for 1000/2000/3000 H_2_O systems. Reproduced with permission [45]. Copyright 2023, Wiley-VCH. (**f**,**g**) Free energy profiles of V oxidation by water under open-circuit and U = 0 V. IS/TS/FS structures are shown above. (**h**) Schematic of potential-dependent MXene oxidation in water. (**i**) Key oxidation steps. Atom colors: V (purple), C (brown), O (red), H (white). Reproduced with permission [47]. Copyright 2024, Spring Nature.

Wu et al. [44] employed first-principles molecular dynamics (FPMD) simulations to reveal that water attack on the Ti_3_C_2_O_2_ surface proceeds through four distinct stages (Figure 3a,b):

① Reversible adsorption stage: Initially, water molecules are randomly distributed between the layers and adsorb onto the surface Ti atoms. At this time, the distance between the water molecule and the Ti atom (Ti-O) min varies between about 2.0–3.5 Å. This distance is close to the sum of the van der Waals radii of Ti and O atoms (3.66 Å), indicating that the adsorption at this time is physical adsorption and the adsorption process is reversible.

② Irreversible adsorption stage: Over time, partial water molecules undergo chemical adsorption on surface Ti atoms, reducing (Ti-O)ₘᵢₙ to 2.0–2.75 Å—close to the sum of their covalent radii (2.26 Å). The strong Ti-water interaction pulls Ti atoms out of their original Ti layer, increasing the z-coordinate of Ti relative to surface O atoms (Ti_z_-max) and inducing slight surface reconstruction, rendering the adsorption irreversible.

③ Water dissociation and Ti atom extraction stage: Ti-polarized water molecules undergo deprotonation, gradually weakening Ti-C bonds until they break—a hydrolysis reaction. The newly formed -OH groups pull Ti atoms toward the surface, further shortening (Ti-O)ₘᵢₙ to ~1.85 Å. Concurrently, Ti-OH groups form and ions are released.

④ Further reaction stage: Once Ti atoms protrude from the surface, they become vulnerable to attack by additional water molecules. Subsequent reactions between water and protruding Ti atoms continuously alter the Ti_3_C_2_O_2_ surface structure, potentially triggering the extraction of more Ti atoms or the formation of other oxidation products.

Hou et al. [45] integrated a deep neural network with an iterative concurrent active learning scheme to develop a neural network potential (NNP) for the V_2_CO_2_-H_2_O system. After 38 iterations, a high-fidelity NNP was obtained, enabling accurate prediction of potential energy. Accelerated molecular dynamics (AlpMD) simulations revealed that the oxidation process of V_2_CO_2_ in water aligns with the first-principles molecular dynamics (FPMD) results reported by Wu et al. [36]: water molecules adsorb onto V atoms to form V-O bonds, followed by O-H bond cleavage of the adsorbed water molecules—assisted by two neighboring molecules—that releases protons and forms stable vanadium oxides. During oxidation, the system’s total energy decreases, indicating that MXene degradation is a spontaneous energy-minimization process (Figure 3c). In ultra-large V_2_CO_2_-H_2_O systems, the oxidation rate significantly declines during the rapid reaction stage as water layer thickness increases (Figure 3d). This stems from two mechanisms: (1) thicker water layers increase the average distance between stable V atoms and water-borne O atoms (Figure 3e); (2) hydrogen-bond networks restrict vertical water molecule movement, reducing contact opportunities with V atoms and inhibiting oxidation. In the saturation stage, water layer thickness influences proton mobility and water binding, thereby modulating the oxidation reaction dynamics. Hou et al. computationally demonstrated that increasing the water layer thickness helps delay the oxidation of V_2_CO_2_ MXene, which aligns with the experimental findings of Wang et al. [46], who utilized inorganic salt hydration to reduce free water and dissolved oxygen in aqueous solutions, thereby slowing oxidation. Storing MXene in a saturated salt solution effectively retards its oxidation. In contrast, MXene stored in pure water for about 30 days exhibits visible whitening (indicating TiO_2_ formation), accompanied by the emergence of TiO_2_ diffraction peaks in XRD patterns, a gradual decrease in UV–vis absorption intensity, and an increase in Ti–O bond intensity in XPS spectra, further confirming the oxidation process. This experimental work strongly validates the findings of Hou et al., confirming that reducing free water molecules delays MXene oxidation. While Wang et al. focused on the hydration effect of inorganic salts to reduce free water, Hou et al. computationally revealed that an increased water layer thickness restricts water molecule mobility, thereby reducing the amount of free water.

Tian et al. [47] employed constant potential ab initio simulations to characterize the oxidation behavior of MXenes from both kinetic and thermodynamic perspectives. V_2_CO_2_ was selected as the primary research object, and an unconstrained AIMD simulation of 50 ps at 300 K was conducted. Oxidation differences between pure water conditions and oxygen-containing environments revealed that dissolved O_2_ in water accelerates MXene oxidation. MD simulations using CP-HS-DM were performed to analyze free energy changes during the reaction under operational conditions. Simulations were conducted in open-circuit (OC) and 0 V electrode potential states to explore the correlation between surface potential and MXene oxidation:

Under the OC state, free energy increased initially, with a reaction energy barrier of 0.451 eV at a distance of 2.456 Å. As the reaction progressed, protons spontaneously dissociated, and free energy dropped to −1.125 eV at 1.66 Å, forming a stable structure (Figure 3f). Under the 0 V potential, the free energy curve exhibited a significant increase, featuring an energy barrier of 0.744 eV (at 2.054 Å) and a final-state free energy of 0.269 eV (at 1.817 Å) (Figure 3g), with only one proton released to form a structure. Low potential hindered the reaction, indicating that MXene oxidation is a potential-regulated electrochemical process (Figure 3h). Extreme potentials influenced water molecule orientation and charge distribution in MXene atomic layers. Under low potential, water molecules adopted a dipole orientation with H atoms pointing toward the substrate plane (H-down), impeding the atomic approach for nucleophilic reaction. Under high potential, water molecules are oriented with O atoms downward (O-down), facilitating an attack on metal atoms. Post-structure formation, water molecules reverted to H-down orientation, hindering subsequent attacks. Low potential also reduced positive charge on the metal layer and enhanced negative charge on functional groups, increasing reaction energy barriers and impeding nucleophilic attack. The nucleophilic attack of water molecules, analogous to the oxygen evolution reaction (OER), represented a critical oxidation step (Figure 3i). In addition to theoretical calculations that can reveal the hydrolysis process of MXene, numerous experimental studies have also elucidated the oxidation process of MXene in aqueous systems. Habib et al. [48]. systematically investigated the effects of air, liquid, and solid environments on the oxidation of Ti_3_C_2_T*_x_* MXene. By exposing vacuum-filtered films to different humidity levels (0%, 50%, and 80%), they demonstrated that increased humidity leads to a rapid decline in electrical conductivity due to the formation of TiO_2_. Jin et al. [49] compared the stability of alkylamine-functionalized (with different chain lengths) and non-functionalized MXene films under high-temperature/high-humidity conditions and in aqueous environments. They observed a significant increase in electrical resistance for non-functionalized MXene films under high humidity, along with a notable enhancement of the TiO_2_ characteristic peak in the Ti 2p XPS spectrum. These studies collectively demonstrate that the oxidation process of MXene accelerates significantly under high humidity and aqueous conditions, leading to irreversible loss of its unique structural and chemical properties.

For MXene-based gas sensors operating under high-humidity conditions, the adverse effects of water molecules are mainly manifested in two aspects. First, water molecules adsorb onto the MXene surface, occupying active sites, increasing electrical resistance, and hindering the adsorption and reaction of target gases. Second, the presence of water molecules induces oxidation of MXene, leading to structural collapse, loss of active sites, blockage of gas diffusion pathways, and an increase in resistance, all of which degrade gas-sensing performance. Oxidation potential was influenced by defects and oxygen-containing species. To improve MXene stability, synthesis methods should be optimized to reduce defects, while functional group engineering serves as an effective strategy. For example, -OH functional groups reduce the positive charge on adjacent metal atoms and act as sacrificial sites, whereas Cl-containing functional groups provide steric shielding via larger atomic radii and reduce metal layer charge density to inhibit oxidation.

## 3. Long-Term Stability Improvement Strategies

**Figure 4:**
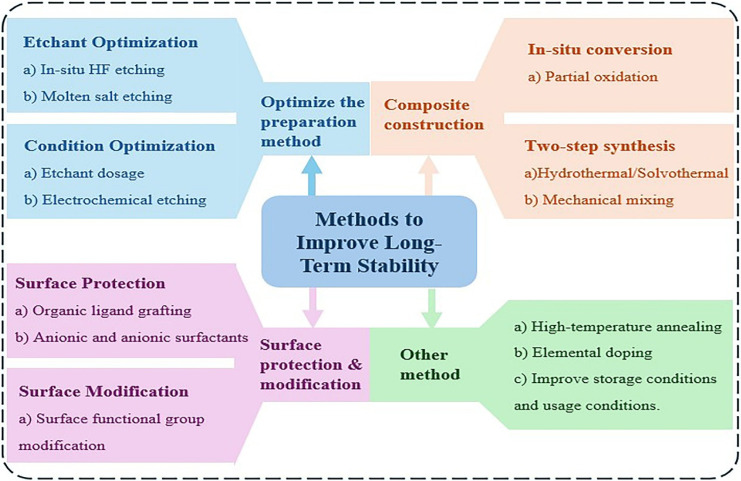
A Comprehensive Overview of Strategies to Enhance the Long-Term Stability of MXene.

To enhance the long-term stability of MXenes, strategies such as optimization of preparation methods, surface protection and modification, composite construction, doping, and high-temperature annealing have been developed (As shown in Figure 4). In this review, these strategies are categorized into four classes, as summarized in the figure. The subsequent sections will introduce these approaches one by one.

### 3.1. Optimize the Preparation Method

Driven by their exceptional properties, MXenes have spurred extensive research endeavors to develop new family members, leading to the continuous expansion of the MXene family and the emergence of diverse preparation methodologies. Current mainstream approaches can be categorized into bottom-up and top-down strategies. The bottom-up method primarily refers to techniques for growing MXenes on substrates [50,51]. For instance, Xu et al. [52] reported the synthesis of large-area, high-quality two-dimensional (2D) ultra-thin α-Mo_2_C crystals via chemical vapor deposition (CVD). These crystals, with a thickness of several nanometers and sizes exceeding 100 μm, exhibit remarkable stability under ambient conditions. In recent years, Yue et al. [53] achieved a one-step vapor-phase synthesis of a few-layer single-phase Ti_2_NCl_2_/Ti_2_CCl_2_, controlling the defect concentration at an extremely low level and effectively enhancing MXene stability. Although such methods can produce large-sized, low-defect MXenes, they suffer from inherent limitations, including high cost, prolonged processing time, and challenges in scalable manufacturing. In contrast, the top-down method predominantly involves selectively etching away the A-element layer from MAX-phase precursors to retain the M_n+1_X_n_ layers [54]. Depending on the etching methods and agents, this can be classified into wet chemical etching, electrochemical etching, alkali etching, and molten salt etching. Despite variations in etching agents and techniques, their underlying etching mechanisms are fundamentally analogous [55].

In the MAX phase, the A-element (e.g., Al in Ti_3_AlC_2_) exhibits a nominal oxidation state approaching zero. The core of the etching process lies in oxidizing atoms to higher oxidation states, such as Al^3+^ or Si^4+^. This oxidation reaction establishes the prerequisites for subsequent ligand binding and ion detachment, with its feasibility dictated by Gibbs free energy. This thermodynamic parameter is influenced by factors including the strengths of M-A and M-X bonds, as well as the Gibbs free energy of byproduct formation. For example, etching Ti_3_SiC_2_ MAX necessitates additional oxidants like HNO_3_ or H_2_O_2_, alongside HF as the -F ligand source [56,57].

**Figure 5:**
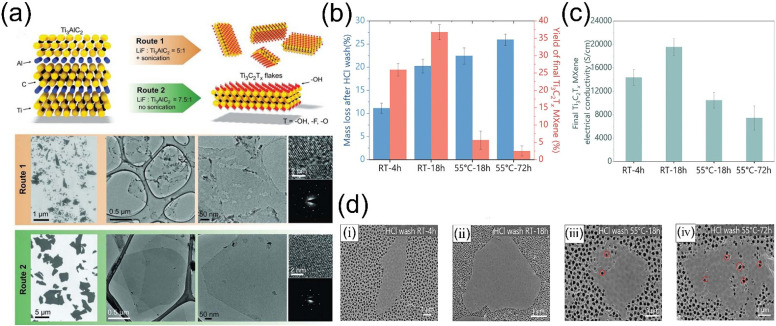
(**a**) Summary of synthetic Routes 1 and 2, with schematic structures of Ti_3_AlC_2_ and Ti_3_C_2_T*_x_*. SEM/TEM images of Ti_3_C_2_T*_x_* flakes via Route 1 and Route 2. Insect: HR-TEM and SAED of Ti_3_C_2_T*_x_* monolayer 2D crystals. Reproduced with permission [62]. Copyright 2016, Wiley-VCH. (**b**) Mass loss of Ti_3_AlC_2_ MAX after HCl pre-wash and corresponding Ti_3_C_2_T*_x_* MXene yield. (**c**) Electrical conductivity of Ti_3_C_2_T*_x_* films. (**d**) SEM images of Ti_3_C_2_T*_x_* MXene flakes synthesized under different pre-etch HCl wash conditions: (i) RT—4 h, (ii) RT—18 h, (iii) 55 °C—18 h, (iv) 55 °C—72 h (The red circle marks a formed defect). Reproduced with permission [61]. Copyright 2023, Wiley-VCH.

Wet chemical etching directly uses HF [58] as the etchant or employs a mixture of fluoride salts (LiF, NH_4_HF_2_, FeF_3_, KF, and NaF) and HCl to generate HF in situ for etching [59,60]. However, fluorine-based etching systems often struggle to produce large-area, low-defect MXene [61], as the presence of defects can induce environmental degradation and reduce MXene stability. Compared to direct HF etching, the situ-formed HF is safer and thus widely adopted. Numerous studies have shown that optimizing the etching process can reduce defects and enhance stability. For example, Lipatov et al. [62] prepared Ti_3_C_2_T*_x_* nanosheets using two methods. Route 1 used the original method: immersing Ti_3_AlC_2_ powder in a LiF-HCl solution (LiF: MAX molar ratio of 5:1), followed by ultrasonic exfoliation. Route 2 was an improved method: increasing the LiF: MAX molar ratio to 7.5:1 and doubling the HCl: LiF ratio, enabling exfoliation without ultrasonication. The nanosheets prepared by Route 2 were larger (4–15 μm), and had cleaner surfaces, more regular edges, fewer obvious defects, better mechanical properties, and more ordered stacking, while those from Route 1 were smaller (200–500 nm), with issues such as incomplete exfoliation, titanium dioxide particles, and pinholes at the edges (Figure 5a). Thakur et al. [61] investigated the effects of factors such as MAX-phase preparation, etching time, and few-layer preparation conditions on the stability of MXene during HF-HCl mixed-acid etching. Using HF-HCl mixed acid at 35 °C for 24 h as the standard method, they studied the impact of different etching times on MXene quality. Longer etching times significantly reduced MXene yield, primarily due to the over-etching of Ti_3_AlC_2_ particles, dissolving smaller MAX particles into nanometer-scale MXene sheets that were easily removed during subsequent centrifugation and filtration steps. The electrical conductivity of MXene was also significantly affected, as shown in Figure 5b,c. As the etching time extended from 24 h to 48 h and then to 72 h, the electrical conductivity of MXene films decreased significantly, because longer etching times introduced more defects into MXene nanosheets (Figure 5d). Increased defects led to higher inter-sheet contact resistance, thereby reducing conductivity.

MXenes etched with different agents exhibit distinct thermochemical properties due to variations in surface chemistry [63]. However, MXenes generally show poor stability at high temperatures, limiting their use primarily to room-temperature gas sensing. Unfortunately, room-temperature MXene-based gas sensors often suffer from baseline drift and poor recovery. In the study by Lee et al. [64], Ti_3_AlC_2_ (MAX phase) was etched using a mixed solution of HCl and LiF to obtain Ti_3_C_2_T*_x_* MXene, which was then subjected to ultrasonic exfoliation to prepare a monolayer dispersion of Ti_3_C_2_T*_x_*. The dispersion was drop-cast onto a flexible polyimide substrate to fabricate a gas sensor. The sensor exhibited p-type responses to all target gases at 100 ppm, with the response magnitudes following the order: ammonia (0.210) > methanol (0.143) > ethanol (0.115) > acetone (0.075). While the sensor demonstrated good stability and reversibility in short-term repeated tests, baseline drift was observed for strongly adsorbing gases such as ammonia. Conversely, Wu et al. [65] employed a solution of NaF + HCl to etch Ti_3_AlC_2_, removing the Al atoms to yield multi-layered Ti_3_C_2_T*_x_*. Through dimethyl sulfoxide (DMSO) intercalation and ultrasonic dispersion, monolayer Ti_3_C_2_ (SL-Ti_3_C_2_) aqueous dispersion was obtained. The sensor fabricated using this monolayer dispersion showed a response of 6.13% to 500 ppm NH_3_, which was four times higher than that to ethanol (1.5%) and ≥0.4% to other gases. During cyclic testing across 50–500 ppm NH_3_ concentrations, the sensor maintained stable responses; however, issues such as prolonged recovery time and baseline drift persisted. To address the challenges of baseline drift and slow recovery in MXene-based sensors operating at room temperature, strategies such as compositing with other materials or noble metal loading have been proposed [66,67]. These approaches will be discussed in detail in the subsequent section on composite construction.

Fluorine-based etching systems pose safety risks to researchers [68] and environmental hazards, prompting a shift toward fluorine-free approaches such as electrochemical etching. This method utilizes circuit voltage to break M–A bonds for selective A-atom etching, typically employing electrolytes like NaCl or HCl [69]. At 0–2.5 V, M–A bonds are cleaved to selectively remove A atoms; higher voltages lead to the removal of M-layer atoms, causing over-etching of MAX into carbide-derived carbon (CDCs) that coat the surface and terminate the etching process. Precise voltage control is therefore critical for successful electrochemical etching [70].

Sheng et al. [71] reported a green fluorine-free approach for fabricating 3D self-supported MXene via anodic electrochemical in situ etching combined with cathodic electrophoretic deposition for electrocatalytic hydrogen evolution. The process involves pressing screened Mo_2_TiAlC_2_ powder into an anodic ceramic disk, using Pt foil as the cathode, performing constant-voltage etching in an NH_4_Cl/LiOH electrolyte, and separating the product by centrifugation. In this electrolyte, Cl^−^ and OH^−^ ions corrode the Al layer, while Li^+^ intercalation expands the interlayer spacing of MXene. At the anode, oxygen evolution and Al etching occur simultaneously; at the cathode, hydrogen evolution and electrophoretic MXene (E-MXene) deposition take place. After multiple hydrogen evolution reaction (HER) cycles, the MXene maintains a stable crystal structure and microstructure without structural collapse or obvious morphological changes, demonstrating robust physical integrity.

Beyond electrochemical etching, molten salt etching emerges as a safe and cost-effective alternative. In 2016, Urbankowski et al. [72] first reported a molten salt method using fluoride salts (59 wt% KF, 29 wt% LiF, 12 wt% NaF), where Ti_4_N_3_T*_x_* powder was mixed with the salts and heated at 550 °C for 30 min under an Ar atmosphere to etch MXene. This approach enables efficient etching without HF and allows tuning of the MXene surface functional group composition [73]. Li et al. [36] developed a general redox-controlled MXene synthesis method in Lewis acid molten salts, involving mixing MAX powder with Lewis acid salts and eutectic salts (e.g., KCl/NaCl) for Ar-assisted etching. Lewis acid cations, with high redox potentials, oxidize the A-site atoms in MAX. By adjusting the MAX/Lewis acid ratios and salt types, this method controls MXene synthesis and surface functional groups, expanding the range of applicable MAX precursors. For instance, MXene etched with CuCl_2_ features ~12 wt% –O and ~14 wt% –Cl terminations—distinct from traditional HF etching (which lacks –OH groups and has higher oxygen content)—thereby significantly influencing material properties.

Functional groups profoundly influence MXene stability and performance. The bonding modes and strengths of –F and =O groups with Ti differ significantly: –F gradually desorbs from Ti_3_C_2_T*_x_* at elevated temperatures, while =O remains stable within specific temperature ranges. –F desorption can induce changes in surface atomic arrangements and structural stability, whereas =O maintains Ti coordination to provide structural support. In aqueous environments, –F is unstable under basic conditions and is readily replaced by –OH, thereby altering surface chemistry and environmental stability [74]. The diversity of surface functional groups also enhances gas selectivity and response: MXenes with specific functional groups exhibit higher sensitivity to target gases due to differing adsorption energies and electronic structures.

### 3.2. Surface Protection and Modification

The edges and surfaces of MXene are highly prone to oxidation when exposed to air and humid environments. To address this, researchers have effectively improved MXene’s stability in operational environments by grafting organic ligands and modifying its surface chemistry. Organic ligand modification not only enhances MXene’s oxidation resistance but also improves gas adsorption and selectivity.

**Figure 6:**
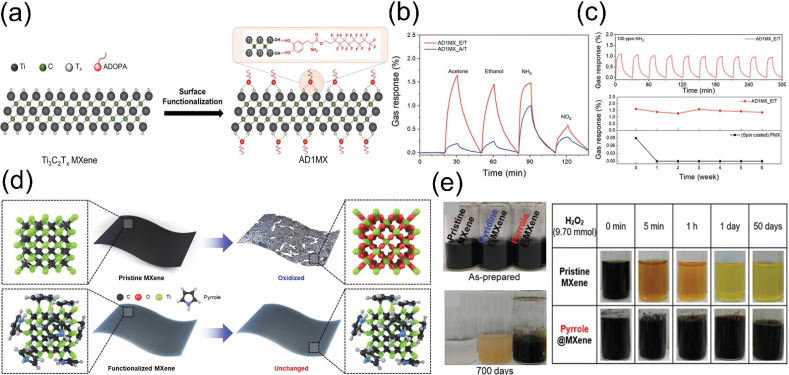
(**a**) Schematic of Ti_3_C_2_T*_x_* MXene functionalization with catechol-based ADOPA molecules. (**b**) AD1MX sensors for detection of acetone, ethanol, NH_3_, and NO_2_. (**c**) Gas response of AD1MX_E/T to repeated 100 ppm NH_3_ injections and stability of AD1MX_E/T vs. pristine Ti_3_C_2_T*_x_* sensors, evaluated by gas response to 100 ppm NH_3_ after 6-week ambient exposure. Reproduced with permission [75]. Copyright 2023, Wiley-VCH. (**d**) Schematic of stability for pristine MXene and heterocyclic aromatic amine (pyridine/pyrrole)-functionalized MXene in water. (**e**) Time-dependent photographs of MXene solutions and optical images of pristine/pyrrole@MXene aqueous dispersions upon adding H_2_O_2_ (9.70 mmol). Reproduced with permission [76]. Copyright 2022, Wiley-VCH.

Creating a hydrophobic protective layer through surface modification is an effective strategy to inhibit oxidation. Kim et al. [75] developed a method to fabricate a series of MXene films using different solvent-nonsolvent combinations, systematically studying their effects on AD1MX film properties. As shown in Figure 6a, AD1MX was dispersed in ethanol (E) or acetone (A) and dropped onto nonsolvents like chloroform (C), dichloromethane (M), or toluene (T). Films prepared with different combinations exhibited similar surface morphologies and high centimeter-scale uniformity. In gas-sensing tests, the AD1MX_E/T film showed the highest response to VOCs—an order of magnitude higher than the pristine sensor (Figure 6b). After exposing AD1MX_E/T and pristine Ti_3_C_2_T*_x_* sensors to ambient conditions for 6 weeks, their responses to 100 ppm NH_3_ were measured: the AD1MX sensor maintained stable, low-fluctuation responses, while the pristine Ti_3_C_2_T*_x_* sensor lost all response after just 1 week (Figure 6c). The superior environmental stability of AD1MX originates from the hydrophobicity of ADOPA end groups, which effectively block oxidants from reaching the MXene surface. Passivating and protecting oxidation-prone edges and defects is another critical approach to enhancing MXene’s environmental stability.

Yun et al. [76] reported a strategy to passivate MXene edges with heterocyclic aromatic amines, enhancing their stability under aqueous conditions. Functionalized MXenes were prepared using pyridine and pyrrole reactions. The stability of pristine MXene, pyridine-decorated MXene (pyridine@MXene), and pyrrole-decorated MXene (pyrrole@MXene) was tested at room temperature and 70 °C, as shown in Figure 6d, with H_2_O_2_ used as a strong oxidant to evaluate their oxidation resistance. At room temperature, pyrrole@MXene maintained its color and 2D morphology for 700 days, while pristine MXene discolored after 30 days and pyridine@MXene after 200 days. In aqueous solutions, pyrrole@MXene remained stable for 6 weeks, whereas pristine MXene and pyridine@MXene decomposed within 3 days and 14 days, respectively (Figure 6e). Under strong oxidation by H_2_O_2_, pyrrole@MXene remained stable in high-concentration H_2_O_2_ (9.70 mmol) for 50 days (Figure 6e), demonstrating exceptional oxidation resistance.

**Figure 7:**
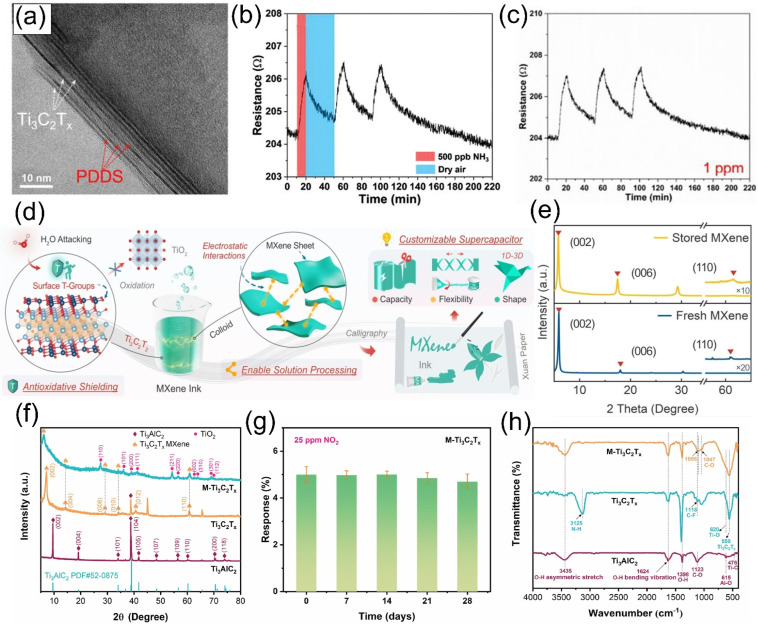
(**a**) TEM image of MP 30%, PDDS (bright layers) is intercalated between the Ti_3_C_2_T*_x_* layers (darker layers). (**b**) repeatability of the sensor at 500 ppb NH_3_. (**c**) The repeatability of the sensor is based on the MP 30% at 1 pp NH_3_. Reproduced with permission [77]. Copyright 2021, Wiley-VCH. (**d**) Schematic illustration showing the in-situ antioxidative shielding of oxyanions, the electrostatic interactions among MXene sheets, and the paper supercapacitors with customizable capacity, flexibility, and shape. (**e**) XRD patterns and Raman spectra of freshly prepared and stored Ti_3_C_2_T*_x_* MXene. Reproduced with permission [81]. Copyright 2024, Wiley-VCH. (**f**) XRD patterns of Ti_3_AlC_2_, Ti_3_C_2_T*_x_*, and M-Ti_3_C_2_T*_x_*, with 2θ ranging from 5 to 80°. (**g**) Long-term (28 days) stability assessment of the M-Ti_3_C_2_T*_x_* sensor for 25 ppm NO_2_. (**h**) FTIR spectra of Ti_3_AlC_2_, Ti_3_C_2_T*_x_*, and M-Ti_3_C_2_T*_x_*. Reproduced with permission [67]. Copyright 2024, Elsevier.

A protective layer composed of hydrophobic materials can not only delay the attack of water and oxygen on the surface of MXene but also improve the gas adsorption and selectivity of MXene, which is beneficial to enhancing the sensitivity and selectivity of sensitive materials. Zhou et al. [77] prepared Ti_3_C_2_T*_x_* /PDDS polymer hybrid materials via microwave-assisted in situ polymerization (TEM images are shown in Figure 7a). The sensor fabricated from the prepared Ti_3_C_2_T*_x_* /PDDS hybrid material exhibited a sensitivity of 2.2% ppm^−1^ to NH_3_ at 500 ppb (Figure 7b). The Ti_3_C_2_T*_x_* /PDDS-based sensor showed acceptable repeatability for continuous exposure to 500 ppb of the gas, with similar responses in three consecutive cycles and a standard deviation of 0.15%. After storing the sensor in a normal indoor environment for 14 days, repeated measurements showed almost identical responses, as shown in Figure 7c, demonstrating the time stability of the sensor. In addition to organic ligands, the use of anionic surfactants and anions such as L-ascorbate [78], citrate [79], and polyanionic salts [80] can also improve the stability of MXene.

Zheng et al. [81] utilized Ti_3_C_2_T*_x_* MXene with anionic oxyanion end groups (Al (OH)_4_^−^). After storage at room temperature for 5 months, the oxyanion-capped Ti_3_C_2_T*_x_* MXene still maintained a well-preserved two-dimensional nanosheet structure and elemental composition, with intact lattice structure and no obvious damage to its chemical structure, as shown in Figure 7d,e. Theoretical calculations reveal that the adsorption energy of oxyanions to molecules is more negative, accompanied by greater charge transfer, and the distance between molecules and surface Ti atoms is farther, effectively inhibiting oxidation. Meanwhile, the oxyanion end groups can alter the electronic structure of MXene, enhance the strength of Ti-C bonds, reduce the oxidation sensitivity of surface Ti atoms, increase surface electron accumulation, and strengthen the electrostatic interactions between MXene sheets. Zhu et al. [82] prepared intercalated materials with excellent electrochemical stability by inserting polyoxometalates (POM) into the interlayers of MXene assisted by quaternary ammonium cations. Cyclic stability tests show that the CV curve of CTAMX-PW12 exhibits almost no change after 20 cycles, and the specific capacitance retention rate reaches 85% after 5000 cycles, far superior to other hybrid materials. Additionally, CTAMX-PW12 demonstrates excellent cyclic stability in both three-electrode and two-electrode systems.

The surface terminations of MXene play a critical role in determining its chemical properties and stability. Adjusting the types of surface terminations is of great significance for optimizing the surface properties and stability of MXene-based materials. Tang et al. [67] prepared M-Ti_3_C_2_T*_x_* MXene with tunable surface oxygen functionalization via a molten salt immersion method. The X-ray diffraction pattern is shown in Figure 7f, and this material was used for detecting NO_2_ at room temperature, significantly enhancing its sensing performance and stability. During 25 days of storage, the response to 25 ppm NO_2_ showed minimal change (as shown in Figure 7g). The M-Ti_3_C_2_T*_x_* MXene was prepared by etching Ti_3_AlC_2_ with mixed acid, followed by high-temperature treatment with KCl and LiCl. Fourier transform infrared spectroscopy revealed that the treated MXene had increased -O functional groups and decreased -F functional groups (as shown in Figure 7h). The difference in surface functional groups is the main reason for the good stability of M-Ti_3_C_2_T*_x_* MXene, as different functional groups have different bonding abilities with the transition metal atoms in MXene, thereby affecting structural stability. Gong et al. [83] prepared iodine-terminated MXene via a Lewis acid molten salt etching method. In cyclic tests, it showed a specific capacitance retention rate of 91% after 100,000 cycles at a current density of 50 A/g, far higher than that of HF-Ti_3_C_2_T*_x_* MXene. After cycling, the iodine termination, Ti-I characteristic peaks, and microstructure of the material showed basically no changes, demonstrating its structural stability. The introduction of iodine terminations enables the energy storage process to utilize the redox reactions of iodine terminations, with reversible changes in the valence state of I elements during charging and discharging. This not only enhances the energy storage performance of MXene but also improves its own stability.

### 3.3. Composite Construction

Compositing MXenes with other materials such as metal oxides, transition metal dichalcogenides, graphite, and conductive polymers can also improve their stability. The combination of MXenes with conductive polymers not only enhances the mechanical properties of MXenes but also improves their selectivity and sensitivity. Commonly used conductive polymers for gas sensors include polyaniline (PANI), poly(3,4-ethylenedioxythiophene): poly(4-styrenesulfonate) (PEDOT: PSS), polythiophene (PTh), and polypyrrole (PPy) [84].

**Figure 8:**
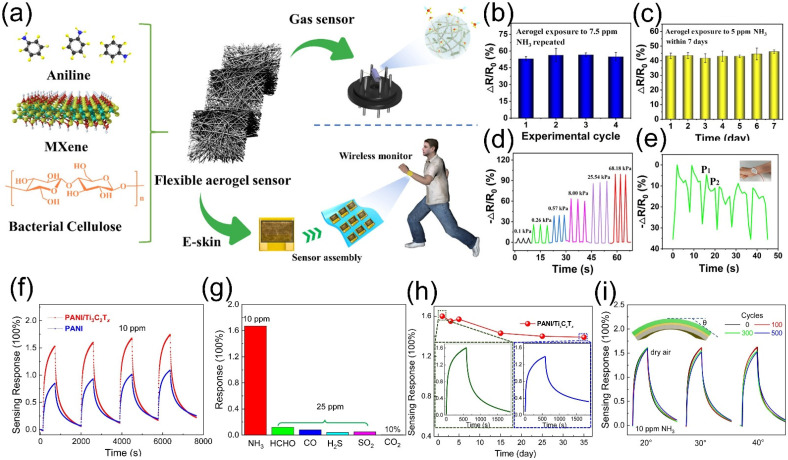
(**a**) Schematics of MXene/PANI/BC aerogel components and their applications for e-skins/gas sensors. (**b**) Response variation of MXene/PANI/BC gas sensor over cycles. (**c**) 7-day stability of the gas sensor. (**d**) Repetitive pressure sensing under loading-unloading cycles. (**e**) Wrist bending sensing response. Insect: sensor attached to the wrist. Reproduced with permission [85]. Copyright 2021, American Chemical Society. (**f**) Reversible response cycles of pure PANI and PANI/Ti_3_C_2_T*_x_* sensors to 10 ppm NH_3_ at RT. (**g**) NH_3_ selectivity against agricultural interference gases at RT. (**h**) Air stability of PANI/Ti_3_C_2_T*_x_* sensor. (**i**) Dynamic NH_3_ sensing response (10 ppm) of PANI/Ti_3_C_2_T*_x_* films under different bending angles (20°, 30°, 40°) and cycles (100, 300, 500). Inset: bending angle definition. Reproduced with permission [86]. Copyright 2020, Elsevier.

Zhi et al. [85] constructed a three-dimensional (3D) network structure via hydrogen bonding interactions, integrating one-dimensional (1D) PANI-bacterial cellulose (BC) nanofibers with two-dimensional (2D) Ti_3_C_2_T*_x_* MXene nanosheets, followed by freeze-drying. This multifunctional architecture enabled integrated pressure and gas sensing, featuring high sensitivity, broad detection range, and wireless transmission capability (Figure 8a). It showed excellent sensitivity to ammonia, with a linear response (R^2^ = 0.997) to 2.5–12.5 ppm NH_3_, a detection limit of 56.49 ppb, and a response retention rate > 80% over 7 days (Figure 8b,c). Additionally, the sensor exhibited a three-stage linear response under 0–70 kPa pressure, with a maximum sensitivity of 327.22 kPa^−1^ (0–3 kPa) capable of real-time monitoring of finger bending, wrist movement, and pulse beats (Figure 8d,e). Li et al. [86] deposited a hybrid membrane on a flexible polyimide (PI) substrate via in situ self-assembly, where PANI and Ti_3_C_2_T*_x_* were combined through electrostatic interactions to form an interpenetrating “nanofiber-sheet” structure. The sensor exhibited a response of 1.5 to 10 ppm NH_3_ and 400% to 50 ppm NH_3_, with a linear range of 0.025–50 ppm (Figure 8f). Its excellent selectivity toward NH_3_ Figure 8g (responses < 10% to interfering gases like HCHO, CO, and H_2_S) originated from hydrogen bonding between NH_3_ and surface functional groups of Ti_3_C_2_T*_x_*, as well as the protonation reaction of PANI. The sensor maintained outstanding stability under short-term repeated testing and mechanical deformation (Figure 8h,i), with an 88% response retention rate after long-term air exposure.

Graphene, as another two-dimensional material, is also a class of materials commonly used for composite construction. Yang et al. [87] reported an in situ sacrificial zinc template-assisted method for preparing 3D porous anti-oxidation MXene/graphene (PMG) composites, addressing MXene agglomeration and stability challenges. During synthesis, zinc powder ionizes into Zn^2+^ under hydrochloric acid, serving as both a cross-linking agent and an in situ sacrificial template/reducing agent to promote self-assembly of MXene and RGO nanosheets into a 3D porous architecture. Electrical conductivity measurements revealed significantly enhanced anti-oxidation capability for the PMG-5 composite while pure MXene experienced severe conductivity decay after 35 days, Zn-MXene showed minimal decline within the first 6 days and remained stable for an additional 54 days. This improvement is attributed to two mechanisms: (1) zinc-mediated reduction and removal of oxygen-containing surface groups on MXene, reducing TiO_2_ nucleation sites; and (2) RGO nanosheets acting as a protective shield to minimize MXene’s exposure to oxygen and moisture, as confirmed by contact angle measurements.

**Figure 9:**
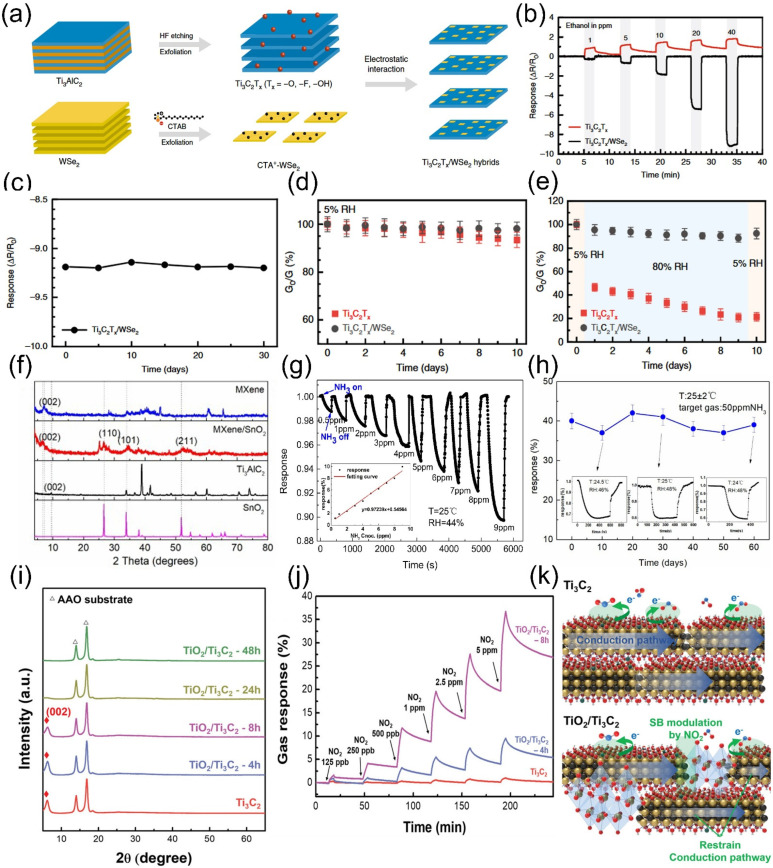
(**a**) Schematic of preparation processes for Ti_3_C_2_T*_x_* /WSe_2_ nanohybrids. (**b**) Real-time sensing response of Ti_3_C_2_T*_x_* and Ti_3_C_2_T*_x_* /WSe_2_ gas sensors to ethanol (1–40 ppm). (**c**) Cycling performance of Ti_3_C_2_T*_x_* /WSe_2_ sensors for 40 ppm ethanol. (**d**,**e**) Electrical conductance changes of pristine Ti_3_C_2_T*_x_* and Ti_3_C_2_T*_x_* /WSe_2_ hybrids under 5% RH and 5–80% RH alternation over 10 days. Reproduced with permission [91]. Copyright 2020, Spring Nature. (**f**) XRD patterns of MXene and MXene/SnO_2_ heterojunctions. (**g**) Response curves of MXene/SnO_2_ sensors to low-concentration NH_3_ (0.5–9 ppm). (**h**) 60-day stability of MXene/SnO_2_ sensors for 50 ppm NH_3_ at RT. Reproduced with permission [99]. Copyright 2020, Elsevier. (**i**) XRD patterns of TiO_2_/Ti_3_C_2_ thick films with controlled oxidation time. (**j**) Real-time gas response of TiO_2_/Ti_3_C_2_ to NO_2_ at varying concentrations. (**k**) NO_2_ sensing mechanisms for Ti_3_C_2_ and TiO_2_/Ti_3_C_2_ films. Reproduced with permission [105]. Copyright 2020, Wiley-VCH.

Two-dimensional transition metal dichalcogenides (TMDs), MXenes, and graphene share the structural feature of two-dimensionality, endowing them with high specific surface area, excellent adsorption properties, abundant redox active sites, and high surface reactivity. Owing to the work function difference, TMDs and MXenes can form heterojunctions to effectively enhance gas sensing performance. Meanwhile, the composited TMDs can act as passivation layers on MXene surfaces, further preventing oxidation caused by external factors such as water molecules and oxygen [88,89,90]. The composite methods generally involve two steps: first etching the MAX phase, followed by integrating TMDs into the MXene surface. Common composite strategies include direct mechanical mixing, hydrothermal/solvothermal methods, solution-based approaches, and in situ synthesis. Chen et al. [91] modified WSe_2_ nanosheets with CTAB cations (CTA^+^) and self-assembled them with Ti_3_C_2_T*_x_* via electrostatic interactions to form hybrids as shown in Figure 9a. The Ti_3_C_2_T*_x_*/WSe_2_ hybrids were deposited on gold interdigitated electrodes on a polyimide (PI) substrate to fabricate sensors (Figure 9a), exhibiting a 12-fold enhancement in response to ethanol compared to pure Ti_3_C_2_T*_x_* (sensitivity increased from 0.02 to 0.24/ppm) with a detection limit as low as 1 ppm (Figure 9b). The sensor demonstrated excellent stability under high humidity and long-term operation (Figure 9c–e). The Schottky junction formed between metallic Ti_3_C_2_T*_x_* and semiconducting WSe_2_ promoted electron transfer, enhancing sensitivity, while the WSe_2_ coating inhibited edge oxidation of Ti_3_C_2_T*_x_*, ensuring superior environmental stability. Zhao et al. [92] prepared double-transition metal MXene (Mo_2_TiC_2_T*_x_*) via HF etching, followed by in situ growth of lace-like MoS_2_ on the edges and surfaces of Mo_2_TiC_2_T*_x_* nanosheets through a hydrothermal method, forming an edge-enriched Mo_2_TiC_2_T*_x_*/MoS_2_ heterostructure. The sensing material showed a response of 415.8% to NO_2_, far exceeding interfering gases like CO_2_, CH_4_, and H_2_S, with excellent selectivity. The sensitivity to 2–50 ppm NO_2_ reached 7.36% ppm^−1^, 123 times higher than single Mo_2_TiC_2_T*_x_* and 24 times higher than MoS_2_, with a detection limit as low as 2.5 ppb. During 6-cycle repeated testing at room temperature, the relative standard deviation (RSD) of responses was <10% and the response attenuation to high-concentration NO_2_ was <14% within 2 weeks, indicating good long-term stability. Han and colleagues hybridized Mo_2_CT*_x_* with MoSe_2_ to develop a composite gas-sensing material. In this system [93], Mo_2_CT*_x_* acts as an electron donor to accelerate charge transfer, while the interfacial defects provide additional adsorption sites for H_2_S. The narrow band gap further enhances electrical conductivity, collectively improving both sensitivity and response speed. The resulting sensor achieves ultrasensitive detection of H_2_S at room temperature, with a response of 629% to 10 ppm and a detection limit of 22 ppb. It also exhibits high selectivity, long-term stability (signal fluctuation < 11% over 40 days), and mechanical flexibility. This work provides an innovative technical solution for real-time oral health monitoring and early screening of periodontitis.

In addition to TMDs, metal oxides such as Fe_2_O_3_ [94], TiO_2_ [95], WO_3_ [96], SnO_2_ [97], and ZnO [98] are also widely used as sensitive materials for composite construction. The hydrothermal/solvothermal method is a relatively straightforward yet effective approach for achieving high-quality composites, making it a popular choice in such applications. The fabrication of metal oxide-MXene composites generally follows a two-step approach: first, etching the MAX phase to obtain MXene, and then integrating metal oxides onto the MXene surface. Common composite strategies encompass direct mechanical mixing, hydrothermal synthesis, solution-based methods, and in situ growth techniques. For example, He et al. [99] utilized this method to develop two-dimensional MXene decorated with SnO_2_ nanoparticles, which was subsequently applied to fabricate an ammonia sensor. This sensor demonstrated exceptional gas-sensing performance toward ammonia at room temperature across a broad concentration range. Notably, the wireless sensor constructed from this composite material exhibited rapid response kinetics and robust stability. The synthesis process entailed two key steps: first, etching Ti_3_AlC_2_ to obtain Ti_3_C_2_T*_x_* MXene, followed by reacting it with tin (IV) chloride pentahydrate (SnCl_4_·5H_2_O) to form a heterojunction structure. X-ray diffraction (XRD) patterns of both the pristine MXene and the MXene/SnO_2_ heterojunction unambiguously confirmed the successful formation of the hybrid material (Figure 9f). Compared with pure MXene-based sensors, the heterojunction-enhanced sensors showcased significantly improved sensitivity, faster recovery behavior, and more stable baseline resistance. Within the low-concentration range of 0.5–9 ppm, the sensor displayed a robust response to NH_3_, achieving low limits of detection (LOD) and quantification (LOQ) (Figure 9g). Furthermore, it demonstrated excellent selectivity, with negligible responses to potential interfering gases such as ethanol, formaldehyde, methanol, and acetone. The sensor’s response to 30 ppm NH_3_ remained highly consistent across five consecutive testing cycles, and long-term stability tests at room temperature over approximately 60 days with 50 ppm NH_3_ revealed that the response fluctuated within a narrow range of 37–42% (Figure 9h), underscoring its exceptional stability. Sun et al. [100] etched Ti_3_AlC_2_ MAX phase with HF, used WCl_6_ as the precursor and ethanol as the solvent to prepare 1D W_18_O_49_ nanorods via solvothermal method. At 300 °C, the W_18_O_49_/ Ti_3_C_2_T*_x_* −2% composite exhibited a response value of 11.6 to 20 ppm acetone, with a detection limit as low as 170 ppb. The response/recovery time for 170 ppb acetone was 5.6/6 s, and the composite showed low responses to interfering gases such as ethanol, formaldehyde, and ammonia. After 100 cycles of repeated testing, no significant response degradation was observed, and the response remained stable under humidity ranging from 60% to 98%. Liu et al. [101] employed a hydrothermal method to etch Ta_4_AlC_3_ for preparing Ta_4_C_3_, which was subsequently composited with NiWO_4_ nanoparticles for humidity sensing. This approach effectively addresses common limitations of MXene-based sensors such as slow recovery and poor linearity. After NiWO_4_ loading, the composite demonstrated a 150-fold enhancement in response compared to pure Ta_4_C_3_, along with excellent stability over 35 days. This developed sensor shows great potential in respiratory monitoring, non-contact sensing, and early diagnosis of diabetes, providing a promising solution for miniaturized humidity sensing platforms.

The in situ oxidation approach for forming oxide composites not only streamlines the compositing process but also imparts additional advantages to MXene through the heterointerfaces generated during oxidation [102,103]. Majhi et al. [104] developed a room-temperature acetone gas sensor based on sea urchin-like V_2_O_5_ hybrid materials derived from V_2_C-T*_x_* MXene, which exhibited exceptional acetone detection performance. By calcining multi-layered V_2_C-T*_x_* MXene at 300–450 °C, a V_2_C-T*_x_*-derived hybrid structure was obtained. Sensors fabricated from this hybrid material and pristine V_2_C-T*_x_* MXene showed significantly enhanced acetone responses at room temperature (23 °C), with the hybrid achieving a detection limit as low as 4.76% for 0.25 ppm acetone. A three-week stability test on the sea urchin-like V_2_C-T*_x_* MXene/V_2_O_5_ sensor revealed negligible response fluctuations demonstrating its outstanding long-term stability and suitability for extended use. Choi et al. [105] employed solution oxidation to generate multiple Schottky barriers (SBs) within Ti_3_C_2_ MXene films (Figure 9i), enabling the fabrication of highly sensitive gas sensors. The interface Schottky barriers in the optimized TiO_2_/Ti_3_C_2_ MXene system enhanced the NO_2_ gas-sensing response by 13.7-fold compared to pristine Ti_3_C_2_ MXene, with a detection limit as low as 125 ppb Figure 9j. Additionally, the in situ formed TiO_2_ acts as a passivation layer to inhibit MXene oxidation, offering a simple and effective strategy to improve sensor performance and stability as shown in Figure 9k.

Noble metal decoration can also improve the stability and gas sensing performance of MXenes, as noble metals enhance selective gas adsorption and catalytic desorption by improving charge carrier transport, reducing recombination, and increasing oxidation resistance. Chen et al. [106] decorated Ti_3_C_2_T*_x_* MXene with gold (Au) nanoparticles via self-reduction, enhancing its adsorption capacity and sensing performance toward volatile organic compounds (VOCs). The Au- Ti_3_C_2_T*_x_* composite exhibited a 3.0% response to 20 ppm formaldehyde with a detection limit of 92 ppb, outperforming pristine Ti_3_C_2_T*_x_* (1.5% response) and graphene (0.85% response). This superior performance arises from the catalytic effect of Au nanoparticles, which promote formaldehyde adsorption and increases active sites, while the high conductivity of Ti_3_C_2_T*_x_* accelerates electron transport. The sensor maintained excellent stability with a response fluctuation of <4.3% over 5 weeks and consistent responses during 5-cycle testing. Similarly, Shilpa M P et al. [107] prepared Ti_3_C_2_T*_x_*-Ag and Ti_3_C_2_T*_x_*-Au composites by mixing AgNO_3_/HAuCl_4_ solutions with Ti_3_C_2_T*_x_* colloids via self-reduction. Among them, Ti_3_C_2_T*_x_*-Ag demonstrated the best gas sensing performance, showing a 0.002–0.01% response to 1–12 ppm SO_2_ at room temperature with response/recovery times of 18 s/12 s at 12 ppm. The sensor also exhibited stable responses during 5-cycle testing.

In addition to compositing with conductive polymers, TMDs, metal oxides, and noble metals, some researchers have also improved the oxidation resistance of MXenes by combining them with other materials. Qian et al. [108] proposed a metal nanoscale armor strategy, sputtering a nanometer-thick copper layer onto the surface of MXene films to form a seamless heterostructure. As the copper layer thickens, the electrical conductivity and oxidation resistance of the MXene@Cu films are significantly enhanced. After 30 days of exposure to air, the MXene@Cu-4 film retains 72.0% of its electrical conductivity, far higher than the 44.3% retention of pure MXene films. Liu et al. [28] developed a Ti_3_C_2_T*_x_* MXene-based composite incorporating extracted bentonite (EB), leveraging EB’s preferential oxygen adsorption and interfacial coupling effects to effectively enhance MXene’s high-temperature oxidation resistance. This composite maintains a stable structure and performance after annealing in air at temperatures above 400 °C for several hours, whereas pure Ti_3_C_2_T*_x_* undergoes severe oxidation under the same conditions.

The heterojunction architecture not only enhances the sensor’s sensitivity and stability but also optimizes MXene’s optoelectronic properties by efficiently capturing light to generate long-lived charge carriers, thereby suppressing electron–hole recombination [109,110,111,112]. Leveraging these unique advantages, our research team has undertaken extensive investigations in this promising research domain.

As previously discussed, the presence of moisture adversely affects the sensing process, particularly for MXene-based gas sensors. However, given the inevitable presence of water vapor during sensor operation, resolving its interference remains a critical challenge. To address this issue, our team designed and fabricated a heterostructure composed of ZnO nanoparticle-pillared Ti_3_C_2_T*_x_* (Ti_3_C_2_T*_x_*/ZnO) [113] This heterostructure effectively promotes the separation of photogenerated electron–hole pairs and enhances charge carrier transport efficiency. The developed sensor exhibits a response of 21.37% to 100 ppm CH_4_ under room temperature, 60% relative humidity, and visible light illumination, demonstrating excellent selectivity and superior performance compared to pure Ti_3_C_2_T*_x_*. Notably, after 150 days of storage in air, the sensor showed only minimal performance degradation and maintained stable phase structure. Unlike conventional sensors whose performance is often compromised by humidity, the Ti_3_C_2_T*_x_*/ZnO sensor exhibits enhanced sensing behavior with increasing humidity (0–80% RH): the response value increases from 16.1% to 21.37%, and remains stable even at 80% RH. This phenomenon is attributed to the fact that humidity, combined with visible light irradiation, promotes the generation of hydroxyl radicals (·OH), rather than inhibiting the sensing reaction. This work provides a feasible pathway for developing highly stable, oxidation-resistant, and humidity-tolerant MXene-based sensors in the future.

**Figure 10:**
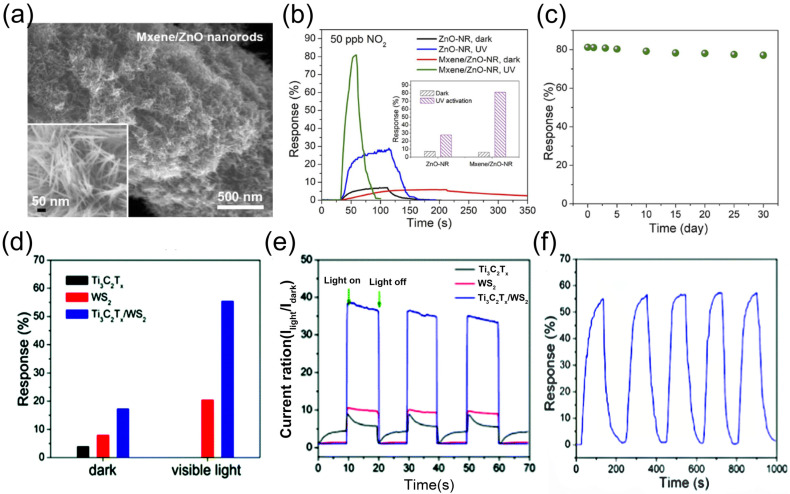
(**a**) MXene/ZnO nanorod hybrids. (**b**) Dynamic response curves and response values (inset) of ZnO-NR and MXene/ZnO-NR sensors to 50 ppb NO_2_. (**c**) 30-day storage stability of sensor responses to 50 ppb NO_2_. (**d**) NO_2_ sensing responses of Ti_3_C_2_T*_x_*, WS_2_ films, and Ti_3_C_2_T*_x_* /WS_2_ hybrid film in dark and under visible-light illumination. Reproduced with permission [108]. Copyright 2021, Elsevier. (**e**) Photoresponse of WS_2_, Ti_3_C_2_T*_x_*, and Ti_3_C_2_T*_x_*/WS_2_ samples. (**f**) Dynamic response curves of Ti_3_C_2_T*_x_*/WS_2_ hybrid film to 2 ppm NO_2_ over 5 cycles. Reproduced with permission [114]. Copyright 2021, The Royal Society of Chemistry.

In addition, our team [114] reported a seed-assisted strategy for fabricating MXene/ZnO nanorod hybrid materials, which exhibit a three-dimensional architecture with a high specific surface area of 146.8 m^2^/g and abundant mesoporous structures, as illustrated in Figure 10a. The sensor based on this hybrid material demonstrates remarkably enhanced response magnitudes and response/recovery kinetics toward 50 ppb NO_2_ under UV irradiation, as shown in Figure 10b. Notably, the sensor achieves an ultra-low detection limit of 0.2 ppb and superior selectivity, with a significantly higher response to NO_2_ compared to other interfering gases. In consecutive four-cycle testing, the sensor maintains stable responses and excellent reproducibility for 50 ppb NO_2_ and retains high responsiveness even after 30 days of air storage (Figure 10c). Under UV illumination, the optoelectronic properties of the MXene/ZnO nanorod hybrid are significantly boosted, featuring improved separation efficiency of photogenerated carriers. Its exceptional gas-sensing performance can be attributed to three key factors: the high-specific surface-area mesoporous structure, the efficient photogenerated carrier separation capability of MXene, and the UV activation effect.

In addition to metal oxides, TMDs have also emerged as effective modifiers for enhancing MXene’s optoelectronic properties [115]. Our team [116] developed a room-temperature visible-light-activated NO_2_ gas sensor based on Ti_3_C_2_T*_x_*/WS_2_ nanocomposites prepared via simple mechanical mixing, showcasing rapid response/recovery kinetics, full reversibility, robust stability, superior selectivity, and ultra-low detection limit. Under visible light illumination, the sensor’s response to 2 ppm NO_2_ at room temperature was significantly amplified to 55.6%—approximately 3.2-fold higher than in the dark (Figure 10d)—with response and recovery times shortened to 56 s and 53 s, respectively. Photoresponse comparisons across three sample configurations revealed that the incorporation of WS_2_ effectively optimized MXene’s optoelectronic behavior (Figure 10e). Gas-sensing evaluations demonstrated that the Ti_3_C_2_T*_x_*/WS_2_ nanocomposite exhibited discernible responses to as low as 10 ppb NO_2_, coupled with excellent selectivity, reversibility, and long-term durability, maintaining consistent performance over five cycles of 2 ppm NO_2_ testing (Figure 10f). This performance enhancement is attributed to the formation of a 2D/2D heterojunction, which synergizes with light activation to improve optoelectronic properties: the heterojunction facilitates efficient separation and transfer of photogenerated carriers, thereby significantly boosting electron–hole pair dissociation efficiency.

### 3.4. Other Improvement Strategies

In addition to the aforementioned methods for improving stability, strategies such as annealing, doping, and optimizing storage conditions have also been explored.

Annealing under an inert atmosphere at appropriate temperatures can significantly enhance MXene stability. For example, Li et al. [117] utilized laser direct writing to fabricate all-solid-state flexible zinc-ion hybrid micro-supercapacitor arrays, with in situ annealing employed to boost their stability. The fabrication process involved preparing single- or multi-layer Ti_3_C_2_T*_x_* MXene via selective etching: large-sized Ti_3_C_2_T*_x_* was spin-coated onto a clean polyimide (PI) substrate as the current collector, patterned by laser direct writing, followed by electrochemical deposition of a zinc anode, spraying of small-sized Ti_3_C_2_T*_x_* as the cathode, coating with a PVA/ZnCl_2_ gel electrolyte, and encapsulation with a PDMS film. The device was then annealed at 300 °C for 30 min under an argon atmosphere. This annealing process effectively removed surface oxygen-containing groups, induced the formation of micro-pores, optimized the structural integrity, and thereby enhanced the cycle stability.

**Figure 11:**
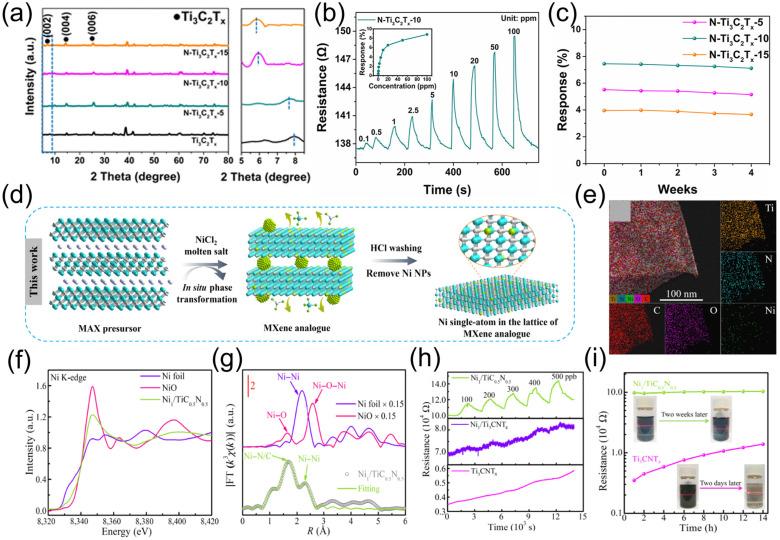
(**a**) XRD patterns of Ti_3_C_2_T*_x_* and N-Ti_3_C_2_T*_x_* nanosheets. (**b**) Dynamic response-recovery curves of N-Ti_3_C_2_T*_x_*-10 sensor to 0.1–100 ppm NH_3_. (**c**) Long-term stability of N-Ti_3_C_2_T*_x_*-10 sensor for 50 ppm NH_3_ at 4% RH. Reproduced with permission [118]. Copyright 2024 American Chemical Society. (**d**) Schematic of single-atom anchoring on MXene analogs via in situ molten salt etching. (**e**) Elemental mapping for structural characterization of Ni_1_/TiN_0.5_C_0.5_. (**f**) XANES spectra and (**g**) k^3^-weighted FT-EXAFS fitting in R space at Ni K-edge. (**h**) Dynamic sensing transients of Ni_1_/TiN_0.5_C_0.5_, Ni_1_/Ti_3_CNT*_x_*, and Ti_3_CNT*_x_* to varying NO_2_ concentrations. (**i**) Resistance changes of Ni_1_/TiN_0.5_C_0.5_ and Ti_3_CNT*_x_* upon air exposure; inset: photographs of their colloidal aqueous solutions under air. Reproduced with permission [126]. Copyright 2022, Spring Nature.

Element doping can also improve the stability and gas-sensing performance of MXene. Fan et al. [118] synthesized nitrogen-doped Ti_3_C_2_T*_x_* (N-Ti_3_C_2_T*_x_*) materials via a one-step hydrothermal method and applied them to fabricate gas sensors. Prepared through a simple one-step hydrothermal process using urea as the nitrogen source, different samples (N-Ti_3_C_2_T*_x_*-5, N-Ti_3_C_2_T*_x_*-10, N-Ti_3_C_2_T*_x_*-15) were obtained by varying the urea dosage. As shown in Figure 11a, the decrease in the 2θ value corresponding to (002) confirms the successful incorporation of nitrogen atoms into Ti_3_C_2_T*_x_*. When tested against different concentrations of ammonia, the N-Ti_3_C_2_T*_x_*-10 sensor demonstrated superior performance. As illustrated in Figure 11b, when testing the sensor with 50 ppm ammonia, its response value reached 7.4%. The response to ammonia was much higher than that to other gases, indicating its high selectivity for ammonia. A long-term stability test of the N-Ti_3_C_2_T*_x_*-10 sensor lasting 4 weeks is shown in Figure 11c, where 50 ppm ammonia was detected in a 4% RH environment. The results show that the sensor’s response value fluctuated minimally throughout the test period, demonstrating stable performance. This indicates that the sensor can maintain good detection performance during long-term use. Nitrogen doping not only enhances stability but also improves the selectivity of Ti_3_C_2_T*_x_* for ammonia because the doping of nitrogen elements increases the carrier density and raises the Fermi level, making electron transfer easier and enhancing the adsorption of ammonia. According to Lewis acid-base theory, it can act as a Lewis acid to form stable complexes with ammonia (a Lewis base), thereby improving the selectivity for ammonia.

Shuvo et al. [119] achieved sulfur doping by mixing and grinding Ti_3_C_2_T*_x_* MXene with thiourea followed by high-temperature heating. Gas-sensing tests revealed that both undoped and sulfur-doped Ti_3_C_2_T*_x_* MXene sensors exhibited significant responses to toluene, with the sulfur-doped sensor showing a stronger response. In the toluene concentration range of 1–50 ppm, the response amplitude was 3–4 times higher than that of the undoped sensor, and it also showed a stable response at a toluene concentration of 500 ppb with a low detection limit. The selectivity of the sensor for toluene originates from the reaction between toluene molecules and adsorbed sulfur species, as well as the characteristics of the benzene ring and methyl group. Cycling and long-term stability experiments demonstrated that the sensor remained stable for at least 1 month.

Conventional doping processes are generally cumbersome, while molten salt etching of MXene can not only achieve regulation of surface functional groups but also enable in situ loading and doping of metal nanoparticles, metal clusters, and single metal atoms. Single metal atoms doped on MXene can act as electron donors to modify the electronic structure of MXene, enhance its gas adsorption capacity, and improve its long-term stability simultaneously [120,121]. During the in situ reduction process, the metastable transition metals in 2D MXene serve as reducing agents to facilitate the formation of chemical bonds between MXene and metals [122,123,124]. Zhang et al. [125] systematically investigated the process of using MXene as an in situ reducing agent for metal reduction in the liquid phase, revealing the nucleation and growth mechanisms during in situ reduction: metals preferentially nucleate at titanium vacancies, and gradually transform from single atoms to nanoclusters and then to nanoparticles as the metal ion concentration increases. The thermodynamics of nucleation is primarily influenced by redox potential and adsorption energy; additionally, the lattice mismatch between metals and the MXene surface affects growth kinetics. Compared with liquid-phase in situ reduction, the molten salt method enables synchronous etching and doping. Chen et al. [126] prepared transition metal nitride (TMN)-supported single metal atom materials for NO_2_ sensing via molten salt etching. Using Ti_3_AlCN as a precursor, Ni_1_/TiC_0.5_N_0.5_ was synthesized through NiCl_2_ molten salt etching and pickling (Figure 11d) was fabricated by dropping Ni (NO_3_)_2_ solution into a Ti_3_CNT*_x_* suspension. EDS mapping images (Figure 11e) show uniform dispersion of Ni species throughout the TiC_0.5_N_0.5_ matrix, with Ni single atoms evenly doping into the TiC_0.5_N_0.5_ lattice to replace Ti sites. Normalized Ni K-edge XANES spectra (Figure 11f) shows an absorption edge position between Ni foil and NiO, indicating that the oxidation state of Ni in Ni_1_/ TiC_0.5_N_0.5_ is between Ni^0^ and Ni^2+^. FT-EXAFS spectra at the Ni K-edge in R-space exhibit distinct peaks at ~1.69 Å and ~2.22 Å (uncorrected for phase shift), attributed to Ni-N/C and Ni-Ni interactions, respectively (Figure 11g), further confirming the doping of Ni single atoms in the TiC_0.5_N_0.5_ lattice. Compared with undoped samples and those doped via traditional two-step methods, molten salt-doped Ni_1_/TiC_0.5_N_0.5_ exhibits superior NO_2_ sensing performance: linear responses to NO_2_ in the range of 10–500 ppb at room temperature (Figure 11h), a detection limit as low as 10 ppb, high selectivity (negligible responses to interfering gases), and stable resistance baselines in both dry and humid environments after multiple tests (Figure 11i). The doped Ni single atoms act as electron donors to increase the electron density of adjacent Ti atoms, promoting NO_2_ adsorption and electron transfer. During molten salt etching, high temperatures cause Ti atoms to detach, creating Ti vacancies. However, Ti vacancies are preferential sites for carbon oxidation: at these sites, C^4−^ readily loses electrons to form amorphous carbon aggregates, while at convex positions such as sheet edges and wrinkles, electron accumulation promotes Ti oxidation. During oxidation, electron–hole accumulation forms an internal electric field that drives Ti cation diffusion, facilitating TiO_2_ nucleation and growth [127]. The strategy of in situ doping via molten salt etching not only achieves etching but also effectively suppresses the formation of Ti vacancies during etching, simultaneously enhancing the stability and gas-sensing performance of MXenes. This provides an effective approach for developing MXene-based gas-sensing materials with high sensitivity, selectivity, and stability.

The Arrhenius equation indicates that the oxidation of MXene proceeds slowly at low temperatures [128]. Additionally, using organic media instead of aqueous media can enhance the oxidation stability of MXene by preventing water interaction and reducing dissolved oxygen in the dispersion medium [129,130]. Therefore, improving storage conditions can also effectively prevent MXenes from oxidation. Roy et al. [131] investigated suitable storage media for Ti_2_CT*_x_* MXene by dispersing it in various organic solvents and water. Samples were stored at room temperature, −20 °C, and −80 °C for 3 months, with dry MXene stored under the same temperature conditions as a control. At room temperature, Ti_2_CT*_x_* MXene fully oxidized in water, partially oxidized in 1-butanol and chloroform, and showed some degradation in the dry state. However, no obvious oxidation or degradation was observed in solvents such as DMSO, DMF, acetone, ethanol, methanol, toluene, n-hexane, and IPA. Analysis of solvent properties revealed that polar solvents with higher surface tension, boiling point, dielectric constant, and viscosity (e.g., DMSO and DMF) were more favorable for long-term storage of Ti_2_CT*_x_* MXene at room temperature. At −80 °C, Ti_2_CT*_x_* MXene dispersed in water showed no oxidation after 3 months; at −20 °C, samples dispersed in IPA also exhibited no oxidation or degradation. In contrast, samples in water at −20 °C showed signs of oxidation. These results indicate that lowering the temperature slows the oxidation or degradation rate of Ti_2_CT*_x_* MXene. The optimal storage method is to disperse it in polar solvents and freeze it at −20 °C or lower, while dry MXene should be stored at subzero temperatures or under freezing conditions. MXene dispersed in water at room temperature begins to degrade within 1 day and shows obvious oxidation after 10 days, so storage in aqueous media at room temperature should be avoided. While organic solvent storage is inconvenient for MXene handling and poses health risks, Wang et al. [46] developed a method using non-toxic, abundant, and low-cost inorganic salts (e.g., NaCl, LiCl, and CaCl_2_) to leverage hydration effects. This reduces the proportion of free water molecules and their interaction with MXene, decreases dissolved oxygen concentration in the solution, and inhibits erosion by free water and dissolved oxygen—thereby extending storage life. MXene can regain good dispersibility and intrinsic properties after rinsing, and the salt protectants can be recycled. This approach offers the advantages of high cost-effectiveness, strong sustainability, and environmental friendliness.

Since the discovery of MXene, the MXene family has continued to expand, with researchers developing various transition metal-based MXenes. The choice of transition metal significantly influences their stability and functional performance.

Kong et al. reported a gas sensor based on monolayer Ta_4_C_3_T*_x_* MXene [132], which exhibits high selectivity and rapid response to ammonia at room temperature. During repeatability tests, the sensor showed no significant variation in response amplitude, demonstrating good reproducibility and reasonable stability. Wu et al. [133]. prepared a stable suspension of Ta_4_C_3_ nanosheets and deposited them onto a melamine sponge through a dip-coating and drying process, constructing a flexible Ta_4_C_3_/melamine sponge piezoresistive sensor. The developed sensor exhibits excellent performance with outstanding stability over 7000 loading-unloading cycles, while maintaining reliable operation across a wide range of temperature and humidity conditions.

Thomas and colleagues fabricated a CO_2_ sensor based on Mo_2_CT*_x_* MXene [134]. Among three tested substrates, the pSi/Mo_2_CT*_x_* composite achieved the best overall performance: a detection limit of 50 ppm at room temperature and excellent stability over 65 days. This system addresses the limitations of conventional sensors, such as large size, high power consumption, and slow response, while the combination of Mo_2_CT*_x_* and pSi offers a feasible pathway toward commercial room-temperature, low-power, high-sensitivity CO_2_ detection devices.

Guo et al. [135]. successfully prepared a defective Cr_2_CT*_x_* MXene sensor via hydrothermal etching combined with N_2_ plasma treatment. The sensor achieved a response of 62.5% toward 10 ppm NO_2_ at room temperature, with a detection limit of 0.1 ppm and excellent stability over 30 days. Its overall performance surpasses most existing MXene and metal oxide-based sensors. Supported by DFT calculations, the study revealed for the first time the synergistic enhancement of NO_2_ adsorption resulting from surface O-functional groups, defect structures, and magnetic moment changes in Cr_2_CT*_x_*, providing theoretical guidance for optimizing the gas-sensing performance of MXene materials.

Ultraviolet irradiation, as an external energy input method, can effectively enhance the sensing performance of metal oxide gas-sensitive materials. However, numerous studies have indicated that for MXene materials, UV exposure accelerates their oxidation.

Habib et al. [48] examined the stability of vacuum-filtered Ti_3_C_2_T*_x_* MXene films under UV light exposure. Their results showed that in ambient atmosphere, just 24 h of UV irradiation led to an over 85% decrease in the electrical conductivity of the MXene film. In contrast, a control sample stored in darkness required 27 days to experience a comparable decline in conductivity. The study suggests that UV light may accelerate MXene oxidation through two possible mechanisms: first, MXene exhibits high absorption of UV light, converting nearly 100% of it into thermal energy, which raises the local temperature and accelerates oxidation kinetics; second, UV light may promote the generation of reactive radicals (such as oxygen and hydroxyl radicals) from ambient oxygen and water molecules, which then attack active sites on the MXene surface (e.g., Ti–C bonds and surface -OH/-O terminal groups), thereby exacerbating structural degradation.

**Figure 12:**
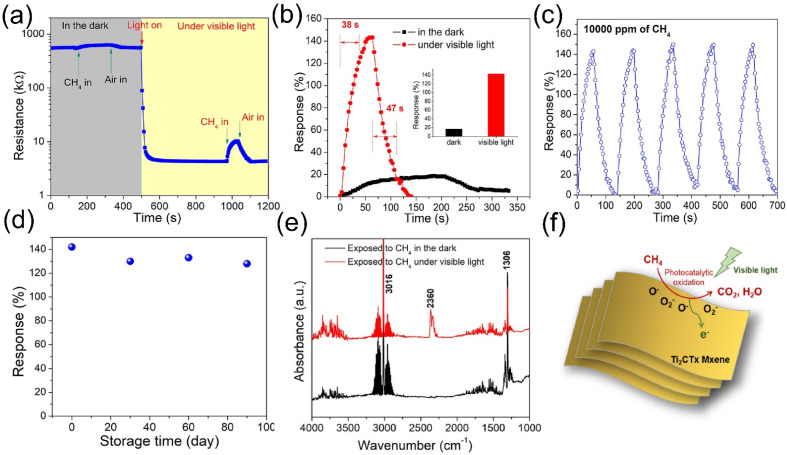
(**a**) Resistance changes kinetics of Ti_2_CT*_x_* sensor to 10,000 ppm CH_4_ under continuous visible-light irradiation (on/off cycles). (**b**) Dynamic response curves and response values of Ti_2_CT*_x_* sensor to 10,000 ppm CH_4_ with/without visible-light irradiation. (**c**) Reproducibility of Ti_2_CT*_x_* sensor for 10,000 ppm CH_4_ (5 cycles). (**d**) Long-term stability of the sensor after air storage. (**e**) In situ DRIFTS spectra of Ti_2_CT*_x_* exposed to 100 ppm CH_4_ in the dark and after 10 min visible-light illumination. (**f**) Schematic of visible-light-induced photocatalysis-enhanced room-temperature CH_4_ sensing by Ti_2_CT*_x_* MXene. Reproduced with permission [138]. Copyright 2021, Elsevier.

Chen et al. [136] further revealed the accelerated degradation and structural evolution of Ti_3_C_2_T*_x_* under UV irradiation. When a colloidal suspension was exposed to a UV light source, the oxidation rate increased significantly compared to the control group. After 24 h of irradiation, the layered structure of MXene completely disappeared, leaving only particulate TiO_2_, whereas the dark-stored control group still retained observable layered structures despite the presence of TiO_2_ particles. Shen et al. [137] also reported that oxidation induced by UV light is significantly more pronounced than that caused by visible light. Their study demonstrated that UV irradiation can completely oxidize Ti_3_C_2_ MXene within 24 h. To mitigate the negative effects of light on MXenes, our team prepared Ti_2_CT*_x_* MXene via mixed acid etching for room-temperature methane sensing [138]. Ti_2_CT*_x_* exhibits a typical layered multi-stack structure, featuring broadband absorption in the visible light region and a narrow bandgap, enabling high photocurrent generation upon light excitation. Leveraging the broadband absorption and high photogenerated carrier efficiency of Ti_2_CT*_x_* MXene in the visible light region, we avoided the use of UV light. As shown in Figure 12a, the resistance of Ti_2_CT*_x_* MXene rapidly decreased under visible light irradiation, accompanied by an enhanced response to methane, demonstrating that visible light irradiation improves the methane oxidation activity of Ti_2_CT*_x_* MXene. This provides a new strategy for the development of room-temperature methane sensors. Under visible light illumination, the Ti_2_CT*_x_* sensor showed a significantly enhanced response to methane, with drastically shortened response/recovery times (Figure 12b). The sensor exhibited concentration-dependent responses within the methane concentration range of 200–10,000 ppm, along with high repeatability, and stability (Figure 12c,d). In situ IR results (Figure 12e) indicate that visible light induces the photocatalytic oxidation of CH_4_ on the Ti_2_CT*_x_* surface, producing CO_2_ and H_2_O.

## 4. Summary and Outlook

This paper synthesizes the current understanding of MXene oxidation and hydrolysis mechanisms, detailing the resulting stabilization strategies and their application in gas sensing. Oxidation primarily initiates at defects and edges, while hydrolysis requires the combined action of water and dissolved oxygen. These degradation processes disrupt MXene’s layered structure and alter its surface functional groups, fundamentally impairing its intrinsic properties. Crucially, the metallic conductivity of pristine MXene degrades into semiconducting behavior upon oxidation/hydrolysis, significantly reducing sensor response and reliability. Furthermore, changes in surface termination groups adversely affect sensor selectivity.

Consequently, future efforts must prioritize developing targeted stabilization strategies—specifically countering oxidation—to preserve MXene properties under environmental stress. Key approaches include:

Optimizing Preparation Methods: Reducing defects is paramount. Early HF etching has evolved towards milder methods like in situ HF generation (HCl/LiF, with ratio optimization), fluoroammonium salts, and fluorohydrogen salts. Notably, molten salt etching (using redox potentials) and electrochemical etching represent significant advances. Molten salt etching offers simplicity, speed, environmental benefits, and reduced defect generation. Critically, it replaces traditional -F, -OH, -O terminations with -Cl, -Br, or -I groups, directly enhancing stability through tailored surface chemistry.

Surface Modification: Organic ligands, anionic surfactants, and polyanionic salts form protective barriers against oxygen/moisture, delaying oxidation. Grafted ligands can also modulate MXene’s electronic structure and gas sensitivity.

Composite Formation: Incorporating metal oxides, graphene, or polymers enhances stability and response. Work function differences enable heterojunction formation, amplifying resistance changes during gas sensing. Controlled oxidation leverages MXene’s inherent transition metals (Ti, V, Cr, Mo) to generate protective metal oxide passivation layers.

Other Strategies: Element doping improves stability, selectivity, and response. Annealing removes interlayer moisture. Storage in organic solvents or inert atmospheres (low temperature, dark) is essential for long-term stability; aqueous storage must be avoided.

Among these, molten salt etching emerges as highly advantageous. It enables simultaneous etching, metal single-atom doping (preferentially occupying Ti vacancies to suppress vacancy-induced degradation), and surface functional group control. This integrated approach concurrently enhances oxidation resistance, modifies the electronic structure, and improves gas adsorption performance. Furthermore, beyond high-temperature requirements, the process poses minimal safety or environmental concerns. Despite these advances, significant challenges remain for MXene in gas sensing.

Research in this area remains limited, with the majority of efforts predominantly concentrated on selectivity or response/recovery times rather than fundamental stability. Crucially, the inherent poor high-temperature stability of MXenes confines most sensors to room-temperature operation, necessitating the development of thermally robust variants. Furthermore, detailed investigations correlating MXene degradation pathways with specific changes in gas-sensing performance are scarce. A comprehensive understanding of this degradation-performance relationship is thus vital for designing truly effective stabilization strategies.

## Data Availability

Data will be made available on request.

## References

[1] Kumar A.N., Pal K. (2022). Amine-Functionalized Stable Nb_2_CT*_x_* MXene toward Room Temperature Ultrasensitive NO_2_ Gas Sensor. Mater. Adv..

[2] Zhou Q., Zeng W., Chen W., Xu L., Kumar R., Umar A. (2019). High Sensitive and Low-Concentration Sulfur Dioxide (SO_2_) Gas Sensor Application of Heterostructure NiO-ZnO Nanodisks. Sens. Actuators B Chem..

[3] Wu Z.-Y., Liu Y.-F., Zhang C., Zheng X.-H. (2025). Electron Sensitization and Chemical Sensitization of ZnWO_4_/WO_3_ Nanorod Heterojunctions for High Performance Triethylamine Sensor. Sens. Actuators B Chem..

[4] Zhang D., Jiang J., Wang T., Li F., Yu H., Dong X., Yang Y. (2024). Flexible Room Temperature Gas Sensor Based on α-Fe_2_O_3_/Ti_3_C_2_T*_x_* MXene Composites for Ppb-Level H_2_S Detection. Sens. Actuators B Chem..

[5] Hermawan A., Zhang B., Taufik A., Asakura Y., Hasegawa T., Zhu J., Shi P., Yin S. (2020). CuO Nanoparticles/Ti_3_C_2_T*_x_* MXene Hybrid Nanocomposites for Detection of Toluene Gas. ACS Appl. Nano Mater..

[6] Shin K.Y., Mirzaei A., Oum W., Kim E.B., Kim H.M., Moon S., Kim S.S., Kim H.W. (2024). Enhanced NO_2_ Gas Response of ZnO–Ti_3_C_2_T*_x_* MXene Nanocomposites by Microwave Irradiation. Sens. Actuators B Chem..

[7] Lin L.-Z., Chen J.-H., Yu Y.-J., Dong G.-H. (2023). Ambient Air Pollution and Infant Health: A Narrative Review. eBioMedicine.

[8] Bai H., Guo H., Wang J., Dong Y., Liu B., Guo F., Chen D., Zhang R., Zheng Y. (2021). Hydrogen Gas Sensor Based on SnO_2_ Nanospheres Modified with Sb_2_O_3_ Prepared by One-Step Solvothermal Route. Sens. Actuators B Chem..

[9] Luo S., Chen R., Wang J., Xiang L. (2023). ZnO/Pd@ZIF-7-Based Gas Sensors for Selective Methane Sensing. ACS Appl. Nano Mater..

[10] Wang J., Hu C., Xia Y., Zhang B. (2021). Mesoporous ZnO Nanosheets with Rich Surface Oxygen Vacancies for UV-Activated Methane Gas Sensing at Room Temperature. Sens. Actuators B Chem..

[11] Li Z., Yao Z., Haidry A.A., Plecenik T., Xie L., Sun L., Fatima Q. (2018). Resistive-Type Hydrogen Gas Sensor Based on TiO_2_: A Review. Int. J. Hydrogen Energy.

[12] Najafi P., Ghaemi A. (2024). Chemiresistor Gas Sensors: Design, Challenges, and Strategies: A Comprehensive Review. Chem. Eng. J..

[13] Hong T., Culp J.T., Kim K.-J., Devkota J., Sun C., Ohodnicki P.R. (2020). State-of-the-Art of Methane Sensing Materials: A Review and Perspectives. TrAC Trends Anal. Chem..

[14] Wang Y., Wang Y., Kuai Y., Jian M. (2024). “Visualization” Gas—Gas Sensors Based on High Performance Novel MXenes Materials. Small.

[15] Zhou J., Wang C., Zhang X., Jiang L., Wu R. (2024). Advances in Two-Dimensional Layered Materials for Gas Sensing. Mater. Sci. Eng. R Rep..

[16] Duan X., Xu D., Jia W., Sun B., Li R., Yan R., Zhao W. (2024). Pt and Black Phosphorus Co-Modified Flower-like WS_2_ Composites for Fast NO_2_ Gas Detection at Low Temperature. Nanoscale.

[17] Govind A., Bharathi P., Mathankumar G., Mohan M.K., Archana J., Harish S., Navaneethan M. (2022). Enhanced Charge Transfer in 2D Carbon- Rich g-C_3_N_4_ Nanosheets for Highly Sensitive NO_2_ Gas Sensor Applications. Diam. Relat. Mater..

[18] Tian W., Wang Y., Zhang Y., Cao J., Guan R.-F. (2021). WO_3_ Nanoflakes Coupled with Hexagonal Boron Nitride Nanosheets for Triethylamine Sensing. ACS Appl. Nano Mater..

[19] Yang C.-R., Cheng P.-W., Tseng S.-F. (2023). Highly Responsive and Selective NO_2_ Gas Sensors Based on Titanium Metal Organic Framework (Ti-MOF) with Pyromellitic Acid. Sens. Actuators Phys..

[20] Kim S.J., Koh H.-J., Ren C.E., Kwon O., Maleski K., Cho S.-Y., Anasori B., Kim C.-K., Choi Y.-K., Kim J. (2018). Metallic Ti_3_C_2_T*_x_* MXene Gas Sensors with Ultrahigh Signal-to-Noise Ratio. ACS Nano.

[21] VahidMohammadi A., Rosen J., Gogotsi Y. (2021). The World of Two-Dimensional Carbides and Nitrides (MXenes). Science.

[22] Zhang S., Meng L., Hu Y., Yuan Z., Li J., Liu H. (2024). Green Synthesis and Biosafety Assessment of MXene. Small.

[23] Xu X., Zhang Y., Sun H., Zhou J., Yang F., Li H., Chen H., Chen Y., Liu Z., Qiu Z. (2021). Progress and Perspective: MXene and MXene-Based Nanomaterials for High-Performance Energy Storage Devices. Adv. Electron. Mater..

[24] Feng C., Ou K., Zhang Z., Liu Y., Huang Y., Wang Z., Lv Y., Miao Y.-E., Wang Y., Lan Q. (2022). Dual-Layered Covalent Organic Framework/MXene Membranes with Short Paths for Fast Water Treatment. J. Membr. Sci..

[25] Yadav M., Kumar M., Sharma A. (2024). Review of Ti_3_C_2_T*_x_* MXene Nanosheets and Their Applications. ACS Appl. Nano Mater..

[26] Kou Y., Hua L., Chen W.-J., Xu X., Song L., Yu S., Lu Z. (2024). Material Design and Application Progress of Flexible Chemiresistive Gas Sensors. J. Mater. Chem. A.

[27] Zhou H., Chen Z., López A.V., López E.D., Lam E., Tsoukalou A., Willinger E., Kuznetsov D.A., Mance D., Kierzkowska A. (2021). Engineering the Cu/Mo_2_CT*_x_* (MXene) Interface to Drive CO_2_ Hydrogenation to Methanol. Nat. Catal..

[28] Liu N., Li Q., Wan H., Chang L., Wang H., Fang J., Ding T., Wen Q., Zhou L., Xiao X. (2022). High-Temperature Stability in Air of Ti_3_C_2_T*_x_* MXene-Based Composite with Extracted Bentonite. Nat. Commun..

[29] Tan A.Y.S., Awan H.T.A., Cheng F., Zhang M., Tan M.T.T., Manickam S., Khalid M., Muthoosamy K. (2024). Recent Advances in the Use of MXenes for Photoelectrochemical Sensors. Chem. Eng. J..

[30] Kruger D.D., García H., Primo A. (2024). Molten Salt Derived MXenes: Synthesis and Applications. Adv. Sci..

[31] Soomro R.A., Zhang P., Fan B., Wei Y., Xu B. (2023). Progression in the Oxidation Stability of MXenes. Nano-Micro Lett..

[32] Cao F., Zhang Y., Wang H., Khan K., Tareen A.K., Qian W., Zhang H., Ågren H. (2022). Recent Advances in Oxidation Stable Chemistry of 2D MXenes. Adv. Mater..

[33] Li J., Chen X., Zhu X., Jiang Y., Chang X., Sun S. (2024). Two-Dimensional Transition Metal MXene-Based Gas Sensors: A Review. Chin. Chem. Lett..

[34] Xia Q., Fan Y., Li S., Zhou A., Shinde N., Mane R.S. (2023). MXene-Based Chemical Gas Sensors: Recent Developments and Challenges. Diam. Relat. Mater..

[35] Lee E., VahidMohammadi A., Yoon Y.S., Beidaghi M., Kim D.-J. (2019). Two-Dimensional Vanadium Carbide MXene for Gas Sensors with Ultrahigh Sensitivity Toward Nonpolar Gases. ACS Sens..

[36] Li Y., Shao H., Lin Z., Lu J., Liu L., Duployer B., Persson P.O.Å., Eklund P., Hultman L., Li M. (2020). A General Lewis Acidic Etching Route for Preparing MXenes with Enhanced Electrochemical Performance in Non-Aqueous Electrolyte. Nat. Mater..

[37] Wang W., Zhou H., Xu Z., Li Z., Zhang L., Wan P. (2024). Flexible Conformally Bioadhesive MXene Hydrogel Electronics for Machine Learning-Facilitated Human-Interactive Sensing. Adv. Mater..

[38] Liu Y., Shi Z., Liang T., Zheng D., Yang Z., Wang Z., Zhou J., Wang S. (2024). The Mechanism of Room-Temperature Oxidation of a HF-Etched Ti_3_C_2_T*_x_* MXene Determined via Environmental Transmission Electron Microscopy and Molecular Dynamics. InfoMat.

[39] Cao W., Nie J., Cao Y., Gao C., Wang M., Wang W., Lu X., Ma X., Zhong P. (2024). A Review of How to Improve Ti_3_C_2_T*_x_* MXene Stability. Chem. Eng. J..

[40] Persson I., Halim J., Hansen T.W., Wagner J.B., Darakchieva V., Palisaitis J., Rosen J., Persson P.O.Å. (2020). How Much Oxygen Can a MXene Surface Take Before It Breaks?. Adv. Funct. Mater..

[41] Mashtalir O., Cook K.M., Mochalin V.N., Crowe M., Barsoum M.W., Gogotsi Y. (2014). Dye Adsorption and Decomposition on Two-Dimensional Titanium Carbide in Aqueous Media. J. Mater. Chem. A.

[42] Yang X., Yao Y., Wang Q., Zhu K., Ye K., Wang G., Cao D., Yan J. (2022). 3D Macroporous Oxidation-Resistant Ti_3_C_2_T*_x_* MXene Hybrid Hydrogels for Enhanced Supercapacitive Performances with Ultralong Cycle Life. Adv. Funct. Mater..

[43] Zhang C.J., Pinilla S., McEvoy N., Cullen C.P., Anasori B., Long E., Park S.-H., Seral-Ascaso A., Shmeliov A., Krishnan D. (2017). Oxidation Stability of Colloidal Two-Dimensional Titanium Carbides (MXenes). Chem. Mater..

[44] Wu T., Kent P.R.C., Gogotsi Y., Jiang D. (2022). How Water Attacks MXene. Chem. Mater..

[45] Hou P., Tian Y., Xie Y., Du F., Chen G., Vojvodic A., Wu J., Meng X. (2023). Unraveling the Oxidation Behaviors of MXenes in Aqueous Systems by Active-Learning-Potential Molecular-Dynamics Simulation. Angew. Chem. Int. Ed..

[46] Wang X., Wang Z., Qiu J. (2021). Stabilizing MXene by Hydration Chemistry in Aqueous Solution. Angew. Chem. Int. Ed..

[47] Tian Y., Hou P., Zhang H., Xie Y., Chen G., Li Q., Du F., Vojvodic A., Wu J., Meng X. (2024). Theoretical Insights on Potential-Dependent Oxidation Behaviors and Antioxidant Strategies of MXenes. Nat. Commun..

[48] Habib T., Zhao X., Shah S.A., Chen Y., Sun W., An H., Lutkenhaus J.L., Radovic M., Green M.J. (2019). Oxidation Stability of Ti_3_C_2_T*_x_* MXene Nanosheets in Solvents and Composite Films. npj 2D Mater. Appl..

[49] Jin Y.H., Han J.-H., Park J., Kim M., Seok S.-H., Chae Y., Sim Y., Seo S., Lee H., Wang J. (2025). Water- and Oxidation-Resistant MXenes for Advanced Electromagnetic Interference Shielding Applications. InfoMat.

[50] Bai W., Shi L., Li Z., Liu D., Liang Y., Han B., Qi J., Li Y. (2024). Recent Progress on the Preparation and Application in Photocatalysis of 2D MXene-Based Materials. Mater. Today Energy.

[51] Liu P., Pan R., Li B., Su Z., Lin B., Tong M. (2024). Mild and Efficient Method for the In Situ Preparation of High-Quality MXene Materials Enabled by Hexafluoro Complex Anion Contained Salts Etching. Adv. Funct. Mater..

[52] Xu C., Wang L., Liu Z., Chen L., Guo J., Kang N., Ma X.-L., Cheng H.-M., Ren W. (2015). Large-Area High-Quality 2D Ultrathin Mo_2_C Superconducting Crystals. Nat. Mater..

[53] Yue F., Xiang M., Zheng J., Zhu J., Wei J., Yang P., Shi H., Dong Q., Ding W., Chen C. (2024). One-Step Gas-Phase Syntheses of Few-Layered Single-Phase Ti_2_NCl_2_ and Ti_2_CCl_2_ MXenes with High Stabilities. Nat. Commun..

[54] Naguib M., Barsoum M.W., Gogotsi Y. (2021). Ten Years of Progress in the Synthesis and Development of MXenes. Adv. Mater..

[55] Zhou C., Zhao X., Xiong Y., Tang Y., Ma X., Tao Q., Sun C., Xu W. (2022). A Review of Etching Methods of MXene and Applications of MXene Conductive Hydrogels. Eur. Polym. J..

[56] Lim K.R.G., Shekhirev M., Wyatt B.C., Anasori B., Gogotsi Y., Seh Z.W. (2022). Fundamentals of MXene Synthesis. Nat. Synth..

[57] Alhabeb M., Maleski K., Mathis T.S., Sarycheva A., Hatter C.B., Uzun S., Levitt A., Gogotsi Y. (2018). Selective Etching of Silicon from Ti_3_SiC_2_ (MAX) To Obtain 2D Titanium Carbide (MXene). Angew. Chem. Int. Ed..

[58] Naguib M., Kurtoglu M., Presser V., Lu J., Niu J., Heon M., Hultman L., Gogotsi Y., Barsoum M.W. (2011). Two-Dimensional Nanocrystals Produced by Exfoliation of Ti_3_AlC_2_. Adv. Mater..

[59] Wang X., Garnero C., Rochard G., Magne D., Morisset S., Hurand S., Chartier P., Rousseau J., Cabioc’h T., Coutanceau C. (2017). A New Etching Environment (FeF_3_/HCl) for the Synthesis of Two-Dimensional Titanium Carbide MXenes: A Route towards Selective Reactivity vs. Water. J. Mater. Chem. A.

[60] Liu F., Zhou A., Chen J., Jia J., Zhou W., Wang L., Hu Q. (2017). Preparation of Ti_3_C_2_ and Ti_2_C MXenes by Fluoride Salts Etching and Methane Adsorptive Properties. Appl. Surf. Sci..

[61] Thakur A., Chandran B.S.N., Davidson K., Bedford A., Fang H., Im Y., Kanduri V., Wyatt B.C., Nemani S.K., Poliukhova V. (2023). Step-by-Step Guide for Synthesis and Delamination of Ti_3_C_2_T*_x_* MXene. Small Methods.

[62] Lipatov A., Alhabeb M., Lukatskaya M.R., Boson A., Gogotsi Y., Sinitskii A. (2016). Effect of Synthesis on Quality, Electronic Properties and Environmental Stability of Individual Monolayer Ti_3_C_2_ MXene Flakes. Adv. Electron. Mater..

[63] Seredych M., Shuck C.E., Pinto D., Alhabeb M., Precetti E., Deysher G., Anasori B., Kurra N., Gogotsi Y. (2019). High-Temperature Behavior and Surface Chemistry of Carbide MXenes Studied by Thermal Analysis. ACS Publ..

[64] Lee E., VahidMohammadi A., Prorok B.C., Yoon Y.S., Beidaghi M., Kim D.-J. (2017). Room Temperature Gas Sensing of Two-Dimensional Titanium Carbide (MXene). ACS Appl. Mater. Interfaces.

[65] Wu M., He M., Hu Q., Wu Q., Sun G., Xie L., Zhang Z., Zhu Z., Zhou A. (2019). Ti_3_C_2_ MXene-Based Sensors with High Selectivity for NH_3_ Detection at Room Temperature. ACS Sens..

[66] Liu Z., He T., Sun H., Huang B., Li X. (2022). Layered MXene Heterostructured with In_2_O_3_ Nanoparticles for Ammonia Sensors at Room Temperature. Sens. Actuators B Chem..

[67] Tang L., Yang H., Wang H., Yang Y., Wang X., Tang G., Zeng D. (2024). Molten Salt-Modified Ti_3_C_2_T*_x_* MXene with Tunable Oxygen-Functionalized Surfaces for Effective Detection of NO_2_ at Room Temperature. Ceram. Int..

[68] Shuck C.E., Ventura-Martinez K., Goad A., Uzun S., Shekhirev M., Gogotsi Y. (2021). Safe Synthesis of MAX and MXene: Guidelines to Reduce Risk During Synthesis. ACS Chem. Health Saf..

[69] Lukatskaya M.R., Halim J., Dyatkin B., Naguib M., Buranova Y.S., Barsoum M.W., Gogotsi Y. (2014). Room-Temperature Carbide-Derived Carbon Synthesis by Electrochemical Etching of MAX Phases. Angew. Chem. Int. Ed..

[70] Wei Y., Zhang P., Soomro R.A., Zhu Q., Xu B. (2021). Advances in the Synthesis of 2D MXenes. Adv. Mater..

[71] Sheng M., Bin X., Yang Y., Chen Z., Que W. (2024). A Green and Fluorine-Free Fabrication of 3D Self-Supporting MXene by Combining Anodic Electrochemical In Situ Etching with Cathodic Electrophoretic Deposition for Electrocatalytic Hydrogen Evolution. Adv. Mater. Technol..

[72] Urbankowski P., Anasori B., Makaryan T., Er D., Kota S., Walsh P.L., Zhao M., Shenoy V.B., Barsoum M.W., Gogotsi Y. (2016). Synthesis of Two-Dimensional Titanium Nitride Ti_4_N_3_ (MXene). Nanoscale.

[73] Li M., Lu J., Luo K., Li Y., Chang K., Chen K., Zhou J., Rosen J., Hultman L., Eklund P. (2019). Element Replacement Approach by Reaction with Lewis Acidic Molten Salts to Synthesize Nanolaminated MAX Phases and MXenes. J. Am. Chem. Soc..

[74] Bashir T., Ismail S.A., Wang J., Zhu W., Zhao J., Gao L. (2023). MXene Terminating Groups O, –F or –OH, –F or O, –OH, –F, or O, –OH, –Cl?. J. Energy Chem..

[75] Kim S., Ko T.Y., Jena A.K., Nissimagoudar A.S., Lee J., Lee S., Oh T., Kang Y.C., In I., Bhattacharjee S. (2024). Instant Self-Assembly of Functionalized MXenes in Organic Solvents: General Fabrication to High-Performance Chemical Gas Sensors. Adv. Funct. Mater..

[76] Yun H., Chae Y., Kim E., Kim H.K., Jang S., Baik M.-H., Ahn C.W., Lee Y. (2022). Ultra-Stable Titanium Carbide MXene Functionalized with Heterocyclic Aromatic Amines. Adv. Funct. Mater..

[77] Zhou J., Hosseini Shokouh S.H., Komsa H.-P., Rieppo L., Cui L., Lv Z.-P., Kordas K. (2022). MXene-Polymer Hybrid for High-Performance Gas Sensor Prepared by Microwave-Assisted In-Situ Intercalation. Adv. Mater. Technol..

[78] Wu C.-W., Unnikrishnan B., Chen I.-W.P., Harroun S.G., Chang H.-T., Huang C.-C. (2020). Excellent Oxidation Resistive MXene Aqueous Ink for Micro-Supercapacitor Application. Energy Storage Mater..

[79] Zhao X., Vashisth A., Blivin J.W., Tan Z., Holta D.E., Kotasthane V., Shah S.A., Habib T., Liu S., Lutkenhaus J.L. (2020). pH, Nanosheet Concentration, and Antioxidant Affect the Oxidation of Ti_3_C_2_T*_x_* and Ti_2_CT*_x_* MXene Dispersions. Adv. Mater. Interfaces.

[80] Natu V., Hart J.L., Sokol M., Chiang H., Taheri M.L., Barsoum M.W. (2019). Edge Capping of 2D-MXene Sheets with Polyanionic Salts to Mitigate Oxidation in Aqueous Colloidal Suspensions. Angew. Chem. Int. Ed..

[81] Zheng Y., Wang Y., Liu D., Zhao J., Li Y. (2025). Unlocking Self-Antioxidant Capability and Processability of Additive-Free MXene Ink towards High-Performance Customizable Supercapacitors. Angew. Chem. Int. Ed..

[82] Zhu J.-J., Gomez-Romero P. (2022). Polyoxometalate Intercalated MXene with Enhanced Electrochemical Stability. Nanoscale.

[83] Gong S., Zhao F., Xu H., Li M., Qi J., Wang H., Wang Z., Fan X., Li C., Liu J. (2022). Iodine-Functionalized Titanium Carbide MXene with Ultra-Stable Pseudocapacitor Performance. J. Colloid Interface Sci..

[84] Sai Bhargava Reddy M., Aich S. (2024). Recent Progress in Surface and Heterointerface Engineering of 2D MXenes for Gas Sensing Applications. Coord. Chem. Rev..

[85] Zhi H., Zhang X., Wang F., Wan P., Feng L. (2021). Flexible Ti_3_C_2_T*_x_* MXene/PANI/Bacterial Cellulose Aerogel for e-Skins and Gas Sensing. ACS Appl. Mater. Interfaces.

[86] Li X., Xu J., Jiang Y., He Z., Liu B., Xie H., Li H., Li Z., Wang Y., Tai H. (2020). Toward Agricultural Ammonia Volatilization Monitoring: A Flexible Polyaniline/Ti_3_C_2_T*_x_* Hybrid Sensitive Films Based Gas Sensor. Sens. Actuators B Chem..

[87] Yang X., Wang Q., Zhu K., Ye K., Wang G., Cao D., Yan J. (2021). 3D Porous Oxidation-Resistant MXene/Graphene Architectures Induced by In Situ Zinc Template toward High-Performance Supercapacitors. Adv. Funct. Mater..

[88] Choi S.-J., Kim I.-D. (2018). Recent Developments in 2D Nanomaterials for Chemiresistive-Type Gas Sensors. Electron. Mater. Lett..

[89] Late D.J., Huang Y.-K., Liu B., Acharya J., Shirodkar S.N., Luo J., Yan A., Charles D., Waghmare U.V., Dravid V.P. (2013). Sensing Behavior of Atomically Thin-Layered MoS_2_ Transistors. ACS Nano.

[90] Liu B., Chen L., Liu G., Abbas A.N., Fathi M., Zhou C. (2014). High-Performance Chemical Sensing Using Schottky-Contacted Chemical Vapor Deposition Grown Monolayer MoS_2_ Transistors. ACS Nano.

[91] Chen W.Y., Jiang X., Lai S.-N., Peroulis D., Stanciu L. (2020). Nanohybrids of a MXene and Transition Metal Dichalcogenide for Selective Detection of Volatile Organic Compounds. Nat. Commun..

[92] Zhao Q., Zhou W., Zhang M., Wang Y., Duan Z., Tan C., Liu B., Ouyang F., Yuan Z., Tai H. (2022). Edge-Enriched Mo_2_TiC_2_T*_x_*/MoS_2_ Heterostructure with Coupling Interface for Selective NO_2_ Monitoring. Adv. Funct. Mater..

[93] Han Q., Hu C., Shi F., Du J., Zhang F., Li C., Wang L., Xu L. (2025). Flexible Mo_2_CT*_x_*/MoSe_2_ Heterostructure Sensors for Ultrasensitive, Room-Temperature Detection of Exhaled H_2_S in Periodontitis Diagnosis. ACS Sens..

[94] Bagherzadeh Enferadi S.M.H., Mirzaei A. (2024). Fe_2_O_3_-Co_3_O_4_ Nanocomposite Gas Sensor for Ethanol Sensing Studies. Ceram. Int..

[95] He X., Zhuang Y., Gao D., Liu H., Zhu J., Huang S. (2025). Leveraging Convolutional Neural Networks for Enhancing Performance of Cs_3_Cu_2_I_5_/TiO_2_ Nanocrystal-Based Carbon Monoxide Gas Sensor. Sens. Actuators B Chem..

[96] Ding J., Xie M., Li Z., Wang Y. (2025). Fabrication of WO_3_ Nanosheets with Hexagonal/Orthorhombic Homojunctions for Highly Sensitive Ozone Gas Sensors at Low Temperature. J. Alloys Compd..

[97] Jia S., Liu Z., Liu W., Liu T., Tian K., Bai S. (2025). Facile One-Step Hydrothermal Synthesis of SnO_2_/SnO p-n Heterostructure Gas Sensor Enables Efficient NO_2_ Detection. Sens. Actuators Phys..

[98] Xia Y., Wang J., Li X., Dan X., Zhou D., Xiang L., Komarneni S. (2016). Nanoseed-Assisted Rapid Formation of Ultrathin ZnO Nanorods for Efficient Room Temperature NO_2_ Detection. Ceram. Int..

[99] He T., Liu W., Lv T., Ma M., Liu Z., Vasiliev A., Li X. (2021). MXene/SnO_2_ Heterojunction Based Chemical Gas Sensors. Sens. Actuators B Chem..

[100] Sun S., Wang M., Chang X., Jiang Y., Zhang D., Wang D., Zhang Y., Lei Y. (2020). W_18_O_49_/Ti_3_C_2_T*_x_* Mxene Nanocomposites for Highly Sensitive Acetone Gas Sensor with Low Detection Limit. Sens. Actuators B Chem..

[101] Liu W., Li M., Feng X., Yin H., Gong S., Yu K., Zhu Z. (2023). Microgram-Level Ta_4_C_3_ Nanosheets Decorated with NiWO4 Nanoparticles as a High-Performance Humidity Sensor. ACS Appl. Nano Mater..

[102] Li H., Zhou Y., Tu W., Ye J., Zou Z. (2015). State-of-the-Art Progress in Diverse Heterostructured Photocatalysts toward Promoting Photocatalytic Performance. Adv. Funct. Mater..

[103] Marschall R. (2014). Semiconductor Composites: Strategies for Enhancing Charge Carrier Separation to Improve Photocatalytic Activity. Adv. Funct. Mater..

[104] Majhi S.M., Ali A., Greish Y.E., El-Maghraby H.F., Mahmoud S.T. (2023). V_2_CT*_x_* MXene-Based Hybrid Sensor with High Selectivity and Ppb-Level Detection for Acetone at Room Temperature. Sci. Rep..

[105] Choi J., Kim Y.-J., Cho S.-Y., Park K., Kang H., Kim S.J., Jung H.-T. (2020). In Situ Formation of Multiple Schottky Barriers in a Ti_3_C_2_ MXene Film and Its Application in Highly Sensitive Gas Sensors. Adv. Funct. Mater..

[106] Chen W.Y., Sullivan C.D., Lai S.-N., Yen C.-C., Jiang X., Peroulis D., Stanciu L.A. (2022). Noble-Nanoparticle-Decorated Ti_3_C_2_T*_x_* MXenes for Highly Sensitive Volatile Organic Compound Detection. ACS Omega.

[107] Shilpa M.P., Ashadevi K.S., Shetty S.J., Bhat S.S., Naresh N., Mishra V., Waikar M.R., Sonkawade R.G., Gurumurthy S.C. (2025). Noble Metal Decorated Ti_3_C_2_T*_x_* MXene for Room Temperature SO_2_ Detection. Sens. Actuators Phys..

[108] Qian W., Si Y., Chen P., Tian C., Wang Z., Li P., Li S., He D. (2024). Enhanced Oxidation-Resistant and Conductivity in MXene Films with Seamless Heterostructure. Small.

[109] Liu Z., Li M., Sun Y., Wang H., Chen H., Tian Y., Wang H., Ding Y., Chen Z. (2023). Integrating Surface and Interface Engineering to Improve Optoelectronic Performance and Environmental Stability of MXene-Based Heterojunction towards Broadband Photodetection. Nano Res..

[110] Li J., Chen S., Ai Z., Zhao X., Li Z., Tian L., Yang Z., Liang H. (2024). Solvothermal Synthesis of CdTiO_3_/Ti_3_C_2_ MXene Composite as a New Efficient Visible Light Photocatalyst. Colloids Surf. Physicochem. Eng. Asp..

[111] Ananda V.R., Ramadhan F.N., Kautsari A.M., Amrillah T., Hermawan A., Yulizar Y., Gunlazuardi J., Sekino T., Orimo S., Yin S. (2025). Powder Engineering of MXene-Based Heterojunction Materials for Photocatalysis and Gas Sensor Applications. Adv. Powder Technol..

[112] Peng C., Yang X., Li Y., Yu H., Wang H., Peng F. (2016). Hybrids of Two-Dimensional Ti_3_C_2_ and TiO_2_ Exposing {001} Facets toward Enhanced Photocatalytic Activity. ACS Appl. Mater. Interfaces.

[113] Chen R., Xia Y., Yang L., He S., Zhao Q., Li X., Wang Y., Gao J., Hou M., Wang M. (2025). Hetero-Engineering-Driven Hydroxyl Radical Generation on ZnO-Pillared MXene Enables Moisture-Tolerant Methane Sensing at Ppm Level. Carbon Future.

[114] Wang J., Yang Y., Xia Y. (2022). Mesoporous MXene/ZnO Nanorod Hybrids of High Surface Area for UV-Activated NO_2_ Gas Sensing in Ppb-Level. Sens. Actuators B Chem..

[115] Abubakr M., Elahi E., Rehman S., Dahshan A., Khan M.A., Rabeel M., Abbas Z., Maqsood M.F., Rehman M.A., Eom J. (2023). Innovations in Self-Powered Nano-Photonics of Emerging and Flexible Two-Dimensional Materials. Mater. Today Phys..

[116] Xia Y., He S., Wang J., Zhou L., Wang J., Komarneni S. (2021). MXene/WS_2_ Hybrids for Visible-Light-Activated NO_2_ Sensing at Room Temperature. Chem. Commun..

[117] Li L., Liu W., Jiang K., Chen D., Qu F., Shen G. (2021). In-Situ Annealed Ti_3_C_2_T*_x_* MXene Based All-Solid-State Flexible Zn-Ion Hybrid Micro Supercapacitor Array with Enhanced Stability. Nano-Micro Lett..

[118] Fan C., Yang J., Ni W., Wu J., Liu X., Li Z., Zhang Y., Quan W., Zeng M., Hu N. (2024). Real-Time and Wireless Transmission of a Nitrogen-Doped Ti_3_C_2_T*_x_* Wearable Gas Sensor for Efficient Detection of Food Spoilage and Ammonia Leakage. ACS Sens..

[119] Shuvo S.N., Ulloa Gomez A.M., Mishra A., Chen W.Y., Dongare A.M., Stanciu L.A. (2020). Sulfur-Doped Titanium Carbide MXenes for Room-Temperature Gas Sensing. ACS Sens..

[120] Li Y., DiStefano J.G., Murthy A.A., Cain J.D., Hanson E.D., Li Q., Castro F.C., Chen X., Dravid V.P. (2017). Superior Plasmonic Photodetectors Based on Au@MoS_2_ Core–Shell Heterostructures. ACS Nano.

[121] Sun Y., Wang Y., Chen J.Y.C., Fujisawa K., Holder C.F., Miller J.T., Crespi V.H., Terrones M., Schaak R.E. (2020). Interface-Mediated Noble Metal Deposition on Transition Metal Dichalcogenide Nanostructures. Nat. Chem..

[122] Zhao D., Chen Z., Yang W., Liu S., Zhang X., Yu Y., Cheong W.-C., Zheng L., Ren F., Ying G. (2019). MXene (Ti_3_C_2_) Vacancy-Confined Single-Atom Catalyst for Efficient Functionalization of CO_2_. J. Am. Chem. Soc..

[123] Pandey R.P., Rasool K., Madhavan V.E., Aïssa B., Gogotsi Y., Mahmoud K.A. (2018). Ultrahigh-Flux and Fouling-Resistant Membranes Based on Layered Silver/MXene (Ti_3_C_2_T*_x_*) Nanosheets. J. Mater. Chem. A.

[124] Lan L., Jiang C., Yao Y., Ping J., Ying Y. (2021). A Stretchable and Conductive Fiber for Multifunctional Sensing and Energy Harvesting. Nano Energy.

[125] Zhang Q., Wang J., Yu Q., Li Q., Fan R., Li C., Fan Y., Zhao C., Cheng W., Ji P. (2024). Metal/MXene Composites via in Situ Reduction. Nat. Synth..

[126] Chen W., Li P., Yu J., Cui P., Yu X., Song W., Cao C. (2022). In-Situ Doping Nickel Single Atoms in Two-Dimensional MXenes Analogue Support for Room Temperature NO_2_ Sensing. Nano Res..

[127] Xia F., Lao J., Yu R., Sang X., Luo J., Li Y., Wu J. (2019). Ambient Oxidation of Ti_3_C_2_ MXene Initialized by Atomic Defects. Nanoscale.

[128] Liaw B.Y., Roth E.P., Jungst R.G., Nagasubramanian G., Case H.L., Doughty D.H. (2003). Correlation of Arrhenius Behaviors in Power and Capacity Fades with Cell Impedance and Heat Generation in Cylindrical Lithium-Ion Cells. J. Power Sources.

[129] Maleski K., Mochalin V., Gogotsi Y. (2017). Dispersions of Two-Dimensional Titanium Carbide MXene in Organic Solvents. Chem. Mater..

[130] Murali G., Reddy Modigunta J.K., Park Y.H., Lee J.-H., Rawal J., Lee S.-Y., In I., Park S.-J. (2022). A Review on MXene Synthesis, Stability, and Photocatalytic Applications. ACS Nano.

[131] Roy C., De S.K., Banerjee P., Pradhan S., Bhattacharyya S. (2023). Investigating Suitable Medium for the Long-Duration Storage of Ti_2_CT*_x_* MXene. J. Alloys Compd..

[132] Kong D., Huang P., Qin F., Liu J., Lin J., Lin Y., Huang H., Wang W., Han C., Zhang S. (2024). Exploring Monolayer Ta_4_C_3_T*_x_* MXene for Quick Ammonia Detection at Room Temperature. Mater. Lett..

[133] Wu F., Meng X., Liu Z., Lv T., Yu L., Zhang J., Zhao Y., Zhao C., Xing G. (2024). Ta4C3 Nanosheet/Melamine Sponges with High Sensitivity and Long-Term Stability for Wearable Piezoresistive Sensors. ACS Appl. Nano Mater..

[134] Thomas T., Ramos Ramón J.A., Agarwal V., Méndez A.Á.-, Martinez J.A.A., Kumar Y., Sanal K.C. (2022). Highly Stable, Fast Responsive Mo_2_CT*_x_* MXene Sensors for Room Temperature Carbon Dioxide Detection. Microporous Mesoporous Mater..

[135] Guo L., Han H., Wang J., Wang P., Du C., Wang B., Yuan Q., Zhai Y., Zhang C. (2024). Defective Cr_2_CT*_x_*-Based Sensors with High Sensitivity for NO_2_ Detection at Room Temperature. J. Mater. Chem. A.

[136] Chen L., Wakeel M., Haq T.U., Chen C., Ren X. (2022). Insight into UV-Induced Simultaneous Photocatalytic Degradation of Ti_3_C_2_T*_x_* MXene and Reduction of U(VI). J. Hazard. Mater..

[137] Shen S., Ke T., Rajavel K., Yang K., Lin D. (2020). Dispersibility and Photochemical Stability of Delaminated MXene Flakes in Water. Small.

[138] Wang J., Xu R., Xia Y., Komarneni S. (2021). Ti_2_CT*_x_* MXene: A Novel p-Type Sensing Material for Visible Light-Enhanced Room Temperature Methane Detection. Ceram. Int..

